# Efficacy and safety of phospholipid nanoparticles (VBI-S) in reversing intractable hypotension in patients with septic shock: a multicentre, open-label, repeated measures, phase 2a clinical pilot trial

**DOI:** 10.1016/j.eclinm.2024.102430

**Published:** 2024-01-29

**Authors:** Cuthbert Simpkins, Michael Moncure, Heather Klepacz, Kristopher Roach, Sadia Benzaquen, Luis Diaz-Caballero, Jonathan Cohen, Daniel Haase, Mukesh Kumar, Harven DeShield, Anthony Manasia, Juan Rodriguez, Prashanth Anamthathmakula, Nik Hurt, Bhaswati Mukherjee, Krishna Talluri

**Affiliations:** a Department of Surgery, University of Missouri Kansas City School of Medicine, Kansas City, MO, USA; b University Health Truman Medical Center, Kansas City, MO, USA; c Department of Critical Care Medicine, Dignity Health Medical Group, Chandler, AZ, USA; d Department of Pulmonary, Critical Care and Sleep Medicine, Einstein Healthcare Network, Philadelphia, PA, USA; e Department of Internal Medicine, Adventist Healthcare Network, Rockville, MD, USA; f Department of Critical Care, R Adams Cowley Shock Trauma Center, Baltimore, MD, USA; g Vivacelle Bio, Inc., Kansas City, MO, USA; h Department of Medicine and Surgery, Icahn School of Medicine at Mount Sinai, New York, NY, USA; i Department of Basic Sciences, University of Health Sciences and Pharmacy, St. Louis, MO, USA; j Sigmadelta Consultancy, Bangalore, Karnataka, India

**Keywords:** Sepsis, Hypotension, Phospholipid nanoparticles, Nitric oxide, Multiple organ dysfunction syndrome

## Abstract

**Background:**

Since the 1990’s attempts to favorably modulate nitric oxide (NO) have been unsuccessful. We hypothesized that because NO is lipophilic it would preferentially localize into intravascularly infused hydrophobic nanoparticles, thereby reducing its bioavailability and adverse effects without inhibiting its production. We aimed to determine the efficacy and safety of intravenous infusion of a fluid comprised of hydrophobic phospholipid nanoparticles (VBI-S) that reversibly absorb NO in the treatment of hypotension of patients in severe septic shock.

**Methods:**

This is a multicentre, open-label, repeated measures, phase 2a clinical pilot trial done at six hospital centers in the USA. Patients in severe septic shock were enrolled after intravenous fluid therapy had failed to raise mean arterial blood pressure (MAP) to at least the generally accepted level of 65 mmHg, requiring the use of vasopressors. The primary endpoint of this study is the proportion of patients in whom MAP increased by at least 10 mmHg. VBI-S was administered intravenously to patients as boluses of 100 ml, 200 ml, 400 ml, and 800 ml at 999 ml/min until the blood pressure goal was reached after which the infusion was stopped, and the MAP was recorded. All patients who received any volume of VBI-S were included in the primary and safety analysis. The study is registered with ClinicalTrials.gov, NCT04257136.

**Findings:**

Between February 17, 2020 and January 3, 2023, 20 eligible patients were enrolled in the study. In all 20 (100%) patients, the goal of increasing MAP by at least 10 mmHg using VBI-S was achieved (p = 0.0087, effect size = 0.654). Mean VBI-S volume required to meet the primary goal was 561.0 ± 372.3 ml. The goal of lowering vasopressor dose was also achieved (p = 0.0017). Within 48 h or less after VBI-S, there was a statistically significant improvement in oxygenation, serum creatinine, clotting variables, procalcitonin, lactic acid, and the sequential organ failure assessment (SOFA) score. At 24 h and 48 h following administration of VBI-S, 12/15 (80%) and 9/12 (75%) patients developed hyperlipidemia, respectively. No severe adverse events of VBI-S were observed, and there were no treatment-related deaths.

**Interpretation:**

These preliminary findings suggest the safety and efficacy of VBI-S in treating hypotension in patients with septic shock. However, a definitive mortality benefit cannot be demonstrated without a randomized controlled study.

**Funding:**

The Naval Medical Research Command—Naval Advanced Medical Development program via the 10.13039/100021976Medical Technology Enterprise Consortium.

**Research in context**

**Evidence before this study**

We searched PubMed for publications using the search terms “nitric oxide synthase”, “selective nitric oxide inhibitor” with the words “clinical trial sepsis” without any temporal or language restrictions. In two trials there was an increase in mortality in septic shock patients. We did not find any clinical trials of selective nitric oxide synthase inhibitors.

**Added value of this study**

Our preliminary findings suggest that VBI-S, a phospholipid nanoparticle formulation, favourably modulates nitric oxide, and shows safety and potential efficacy in the treatment of hypotension and multiple organ failure associated with relative hypovolemia (vasodilatory shock) in patients with severe septic shock. No severe adverse effects associated with VBI-S were observed.

**Implications of all the available evidence**

This study supports the possibility that VBI-S may improve the efficacy of treatment of patients who are hypotensive due to sepsis, in which overproduction of nitric oxide plays a significant role. However, further randomised controlled trials are needed to verify our findings.

## Introduction

The explosion of new knowledge of the pathophysiology of sepsis over the past 40 years has resulted in only modest improvements in treatment.^1^, ^2^, ^3^, ^4^, ^5^, ^6^, ^7^ One impactful improvement was the initiation by Rivers et al.^8^ of early intravenous fluid therapy to address absolute hypovolemia due to reduced intravascular volume that occurs in sepsis. However, there are patients whose hypotension is not responsive to fluid. Vasopressors are then deployed to elevate blood pressure. But the vasculature is hyporesponsive to vasopressors and eventually may become unresponsive. A key element is excessive nitric oxide that contributes to a state of relative hypovolemia marked by vasodilation^9^ and depressed cardiac contractility.^10^ Currently, no treatment for relative hypovolemia has emerged. Since NO dysregulation is a significant factor, inhibition of its production would be a logical approach. However, NO is essential to vital functions such as oxidative phosphorylation and the regulation of cardiac contractility.^11^, ^12^, ^13^, ^14^, ^15^ Therefore, inhibition of NO production could have adverse effects. Illustrative of this point, a non-selective nitric oxide synthase inhibitor, inhibitor 546C88 caused increased mortality in a clinical trial.^16^ Negative results of preclinical testing of selective nitric oxide synthase inhibitors are not supportive of a clinical trial.^17^^,^^18^ Scavengers of NO such as hydroxocobalamin and guanylate cyclase inhibitors acting downstream of NO are associated with a high percentage of non-responders, adverse effects and a correlation with increased mortality.^19^, ^20^, ^21^, ^22^, ^23^, ^24^, ^25^, ^26^, ^27^, ^28^, ^29^, ^30^, ^31^, ^32^

The goal of favorably modulating NO is further complicated by the heterogeneity of the microcirculation in which there are regions of increased diameter due to locally elevated concentrations of NO, prostaglandins, hydrogen sulfide or carbon monoxide and decreased diameter due to local factors such as endothelin, thromboxane, capillary occlusion caused by non-deformable erythrocytes, neutrophil adhesion, a procoagulant state and a low regional concentrations of nitric oxide synthase. More variation is introduced by the leakage of fluids and proteins and the production of reactive oxygen species and injurious free radicals of NO such as peroxynitrite in the microcirculation.^33^, ^34^, ^35^ These numerous complex and contradictory actions in which simultaneously increased and reduced diameter exist in the microcirculation, and the essential homeostatic role of NO make it a difficult target for direct therapeutic intervention. An alternative therapeutic intervention, reported by Pititjeans et al.^36^ and Lankadeva et al.^37^ in which sympathetic outflow is inhibited, has produced encouraging preclinical experiments and preliminary clinical observations.

Nonetheless, an approach to the modulation of NO that reduces its bioavailability where it is excessively produced, increases bioavailability where its concentration is insufficient, does not interfere with its production and that is safe would be beneficial. Our strategy using hydrophobic nanoparticles appears to fulfill these requirements. Our studies with hydrophobic nanoparticles began in an animal model of clinical death that we developed in which blood was rapidly removed from mice until they lost spontaneous respiration.^38^^,^^39^ We found that spontaneous respiration and a viable blood pressure could be restored if we rapidly infused Ringer’s lactate intra-arterially (IA) via the carotid artery. In contrast, Ringer’s lactate infusion via the jugular vein did not result in reanimation. Further experimentation showed that phospholipid nanoparticles like VBI-S were far more effective in elevating blood pressure and survival after IA infusion compared to Ringer’s lactate. Histological studies showed no injury to the lungs due to replacement of 55% of the blood volume with phospholipid nanoparticles.^38^ Additional studies in our laboratory showed that there was no injury to any other organs we examined including brain, heart, kidneys, intestine, spleen, or liver. Because NO is lipophilic, we hypothesized that it would be absorbed and released by the hydrophobic nanoparticles in VBI-S leading to reduction of NO in regions of excessive production and delivery to regions of insufficient concentration. This would lead to elevation of blood pressure and improved microcirculation. Given that oxygen is a lipophilic gas we hypothesized that oxygen also would be taken up and delivered by VBI-S. We, therefore, measured the uptake and release of oxygen and nitric oxide using mass spectroscopy and chemiluminescence.^38^, ^39^, ^40^

We found that the oxygen and NO content of the phospholipid nanoparticles was as predicted by calculations using the oil/water partition coefficient of these gases, suggesting that there was no loss of either gas by interacting with VBI-S. Based on these preclinical data and already existing extensive literature in support of the safety of these phospholipid nanoparticles the FDA did not require that Vivacelle Bio do a phase I clinical trial of our formulation of phospholipid nanoparticles, VBI-S, in septic shock patients.^42^, ^43^, ^44^, ^45^, ^46^, ^47^, ^41^ We then proceeded to an initial phase IIa study of 20 patients to assess the variability of the response to VBI-S to facilitate planning for a subsequent definitive study.

Because VBI-S is a colloid and the favorable results of our preclinical experiments in rapid severe blood loss, we concluded that VBI-S would be effective in absolute hypovolemia in which hypotension occurs because of blood loss in addition to relative hypovolemia in which hypotension is caused largely by NO-induced vasodilation. The hydrophobic nanoparticles are comprised of soybean oil containing micelles, liposomes comprised of phosphatidylcholine, and sodium chloride that have a mean diameter of 50-nm as determined by electron microscopy. As shown in Supplementary Figure S1 and Supplementary Table S1 of the supplementary section we found that VBI-S absorbed 100% more NO than water and 45% more oxygen than water. Both gases were readily released from VBI-S.

Because of individual variability in the amount of NO produced we titrated the volume of VBI-S given to patients to a target blood pressure. This is consistent with the administration of fluid therapy in general. This mechanism has not yet been proven in vivo. Nonetheless, it is the hypothesis that led to this clinical trial of VBI-S.

We tested this concept in patients who had severe septic shock in whom standard fluid therapy had failed to elevate their mean arterial pressure (MAP) to ≥65 mmHg, the generally accepted level. The primary endpoint was to determine if VBI-S infusion elevated MAP by at least 10 mmHg. The secondary endpoint was to reduce the dose of vasopressors. Determination of the effect of VBI-S on organ function and the Sequential Organ Failure Assessment Score (SOFA score) were exploratory or tertiary endpoints.

## Methods

### Study design

This is a multicentre, open-label, repeated measures, phase 2a clinical pilot trial done at six hospital centers in the USA. The protocol for this trial is available in the Supplementary Materials. The sites for the study were; University Health Truman Medical Center in Kansas City, MO; Chandler Regional Medical Center, Chandler AZ; Jefferson Einstein Hospital, Philadelphia, PA; Shady Grove Adventist Hospital, Rockville, MD; R Adams Cowley Shock Trauma Center, Baltimore, MD and The Mount Sinai Hospital, New York, New York. The study protocol was approved by the IRB of each participating site after which approval was obtained from the Western Institutional Review Board (WIRB) and Copernicus Group (WCG-IRB; https://research.wayne.edu/irb/wirb; reference number 20192983). After this approval, the study was approved by the Office of Human Research Oversight (OHRO) of the United States Department of Defense. Informed consent prior to enrollment and intervention was given by legally authorized representatives since none of the patients was able to provide consent for themselves. Vivacelle Bio, Inc. (Kansas City, MO) is the manufacturer of VBI-S. This study is in compliance with the CONSORT checklist for reporting a feasibility or pilot study.

In each patient MAP had decreased below 65 mmHg and the infusion of standard fluids (Ringer’s lactate, normal saline, 5% albumin or 25% albumin) had failed to elevate MAP to at least 65 mmHg. This failure to sufficiently elevate MAP led to the administration of vasopressors to attempt to achieve the blood pressure goal. VBI-S was infused to elevate MAP after the failure of fluid therapy and the initiation of vasopressors.

No fluid comparator was used because it is well-established that after standard fluids have failed to elevate blood pressure, further administration of these fluids with a goal of achieving an increase in MAP by 10 mmHg would lead to the rapid deterioration of the patient. The administration of standard fluids in this study did not adhere to the SSC guideline that requires fluid infusion at 30 ml/kg over 3 h. It was left up to each individual intensivist to determine how they would resuscitate their patient. It is being increasingly recognized that protocols mandating that fluid to reach a MAP of 65 mmHg at 30 ml/kg within 3 h or 2000 ml over a defined time would be harmful to some patients.^48^ Therefore, the volume of fluid used in an attempt to elevate MAP to at least 65 mmHg was individualized. Because the volume of fluid needed in the attempt to raise blood pressure was highly variable and specific to the patient it was decided that no required volume would be imposed, and the volume of fluid given prior to starting vasopressors should be the decision of the patient’s physician. To determine efficacy, each patient was compared to their own control with variables measured before and after VBI-S.

Laboratory and bedside tests were done within 6 h prior to infusion of VBI-S and 24 ± 6 h and 48 ± 6 h after the completion of the initial VBI-S dose. These tests included arterial blood gas (pH, PaO_2_, pCO_2_, HCO_3_, base excess), FiO_2_, oxygen saturation, lactic acid, Na, K, Cl,HCO_3_, BUN, creatinine, glucose, aspartate amino transferase, alanine amino transferase, alkaline phosphatase, amylase, lipase, bilirubin (total, direct, indirect), troponin, procalcitonin, triglycerides, international normalized ratio, prothrombin time, partial thromboplastin time, white blood cell count, hematocrit, hemoglobin and platelet count.

After the 48-h treatment period patients were followed weekly for 28 days, until hospital discharge or expiration. Every week a physical exam was done, and the laboratory tests mentioned above were done except the blood gas after the removal of the arterial line. The SOFA score was also calculated if the arterial line was in. The ratio of the partial pressure of arterial oxygen (PaO_2_) over the fraction of inspired oxygen (FiO_2_) was calculated unless the patient was no longer on the ventilator or did not have an arterial line.

### Patients

Patients who were improving on standard therapy were not enrolled. Unlike other septic shock studies, there was no exclusion for patients expected to die within 12 h or hemodynamic or respiratory instability. There was no lower limit of cardiac index and no limitation on the length of time that the patient had been on vasopressors, or on the severity of respiratory dysfunction. Because of the failure of fluid therapy all enrolled patients were on vasopressors at the time that VBI-S was administered. VBI-S was given to the consented patients who were not improving on standard therapy. Standard therapy included antibiotics, fluids, mechanical ventilation, high-flow oxygen, and vasopressors. Twenty patients that met the inclusion and exclusion criteria were enrolled in the study. The inclusion criteria were (1) Male or female at least 18 yrs of age; (2) Evidence of bacterial infection demonstrated by positive blood culture or a known source of infection or an elevated procalcitonin of ≥2 ng/ml; (3) Patient has a mean blood pressure <65 mmHg that is unresponsive to fluids currently available on the market; (4) Sequential Organ Failure Assessment (SOFA) score ≥5; (5) Sepsis diagnosis: The presence of infection which can be proven or suspected by 2 or more of the following criteria: lactate >2 mmol/L, mottled skin, decreased capillary refill of nail beds or skin, fever >38.3 °C, or 101 °F, hypothermia <36 °C core temperature (<36°C), heart rate >90 beats per min., tachypnea Rate >20 cycles per min., change in mental status, significant edema or positive fluid balance (>20 ml/kg over 24 h), hyperglycemia (>140 mg/dL) in someone without diabetes, white blood cell count >12,000 or less than 4000, or with >10% "bands" (immature forms), elevated procalcitonin in serum (≥2 ng/ml), arterial hypoxemia (PaO_2_/FiO_2_ < 300 mmHg), acute drop in urine output (<0.5 ml/kg/h for at least 2 h despite fluid resuscitation, or about 30 ml/h for a 70 kg person), creatinine increase >0.5 mg/dL (44.21 μmol/L), INR >1.5 or aPTT >60 s, absent bowel sounds (ileus), high bilirubin (total bilirubin >4 mg/dL). Exclusion criteria were (1) Patients with a ventricular assist device; (2) acute coronary syndrome; (3) pregnant; (4) bronchospasm; (5) mesenteric ischemia; (6) emergency surgery; (7) acute liver disease (hepatitis B and C as examples); (8) Liver failure with a model for end-stage liver disease (MELD) score ≥19; (9) Hematologic or coagulation disorders including thrombocytopenia (platelet count <50,000) and associated with hemodynamically significant active bleeding; (10) Absolute neutrophil count <1000 mm^3^; (11) Current participation or participation in another experimental or device study within the last 30 days before the start of this study. Patient may be included if on other drugs for COVID-19 and or septic shock; (12) Patients with a known allergy to soybeans or eggs; (13) Patient is hypervolemic as assessed by CVP, ultrasound, Swan Ganz catheter, Flo-Trac, esophageal doppler, bioimpedance, ECHO, partial carbon dioxide rebreathing (NICO), lithium dilution (LIDCO) or other method published in a peer-reviewed journal. Endpoints found in the literature were acceptable for the study. For central venous pressure (CVP) the endpoint was a pressure of 8–12 mmHg.^8^ For ultrasound when the collapsibility index <20% the patient is not considered to be adequately fluid resuscitated.^49^ For Swan Ganz Catheter, Flo Trac, esophageal doppler, bioimpedance, NICO and Lithium dilution methods reaching the plateau of the ventricular function curve is indicative of adequate fluid resuscitation.^50^

### Randomisation and masking

This was an open label study performed in the critical care units of six hospitals. All patients received intravenous VBI-S after the administration of vasopressors. VBI-S is a white fluid that is very different from other IV fluids in appearance. All patients’ legally authorized representatives had knowledge of the treatment assignments after enrollment and throughout the study. None of the patients were aware of their treatment because of the severity of their illness.

### Procedures

Blood pressure was measured via an arterial line during the 48-h treatment period and while in the critical care unit. Prior to VBI-S blood pressure was observed for 15 min to ensure stability. VBI-S was given as increasing boluses of 100 ml, 200 ml, 400 ml, 800 ml at a rate of 999 ml/min. There was a 2-min pause between boluses. Blood pressure was taken immediately before VBI-S and after each stepwise bolus until the MAP increased by 10 mmHg or more. Once the goal of an increase in MAP of 10 mmHg or more was met VBI-S was stopped. The maximum cumulative dose in 24 h was 1500 ml. Vasopressors were not weaned until the goal of increasing MAP by 10 mmHg or more was reached. Weaning of vasopressors was done slowly to maintain a MAP of 60–65 mmHg. After the goal of increasing MAP by 10 mmHg or more had been achieved, more VBI-S was given to the patient PRN as determined by the physician for the 48-h treatment period and beyond using the same dosing sequence. At other times MAP was measured at least every hour for 48 h or as long as the patient was in the Intensive Care Unit (ICU). After the first dose of VBI-S, patients were followed weekly until hospital discharge, death or 28 days whichever came first.

### Outcomes

The primary endpoint of this study is the proportion of patients in whom mean blood pressure increased by at least 10 mmHg with a mean blood pressure target of 60–65 mmHg. Responses were assessed by investigators at each site. The sole secondary endpoint of this study is the proportion of patients in whom the dose of vasopressor drugs could be decreased after infusion of VBI-S to maintain a mean arterial pressure of 60–65 mmHg.

After the blood pressure target was reached, vasopressor dose was weaned while maintaining a MAP of 60–65 mmHg. At the discretion of the patient’s physician more VBI-S was allowed as needed to maintain the MAP of 60–65 mmHg and to assist in the weaning of vasopressors. The same protocol of increasing boluses of VBI-S that had been followed for the initial dose was followed for subsequent doses of VBI-S. Adverse events were assessed continuously from the time of the initial dose of VBI-S until 28 days after the first dose, hospital discharge or death. The flow of patient management is shown in Supplementary Figure S2 of the Appendix.

### Statistical analysis

We decided to do a phase IIa study to determine the variance of the data which would provide guidance for calculating the number of patients needed in a phase III trial. We also conducted a phase IIa trial in order to assess the efficacy of our dosing protocol and the safety of VBI-S. In deciding on the number of patients to enroll in the phase IIa trial we relied upon the precedent of the number of patients in other phase IIa trials of a vasopressor and in trials of fluid therapy.^51^, ^52^, ^53^, ^54^ The change in variables from baseline values was summarized using the mean and the standard deviation. The analysis was on an intention to treat basis. Normality of the data for the primary endpoint was tested using a normal Q–Q plot which is in Supplementary Figure S3 of the Appendix. Since the data failed to follow the normal distribution, the Wilcoxon signed rank test was used to compare baseline data paired to data points obtained at 24 ± 6 h and 48 ± 6 h after the initial dose of VBI-S needed to increase MAP by at least 10 mmHg. Data gathered in subsequent weeks were analyzed similarly. All data for the secondary and tertiary endpoints were not obtained. For these endpoints, the patient numbers at each time point differ because of deaths or missing data that were randomly not obtained by the site. All data were utilized in the statistical analysis without selection.

This study is registered with ClinicalTrials.gov, NCT04257136.

### Role of the funding source

The funder of the study had no role in study design, data collection, data analysis, data interpretation, or writing of the report. CS, MM, HK, KR, SB, LD-C, JC, DH, MK, PA, AM, BM and KT had access to the dataset and had final responsibility for the decision to submit this article for publication.

## Results

**Fig. 1:**
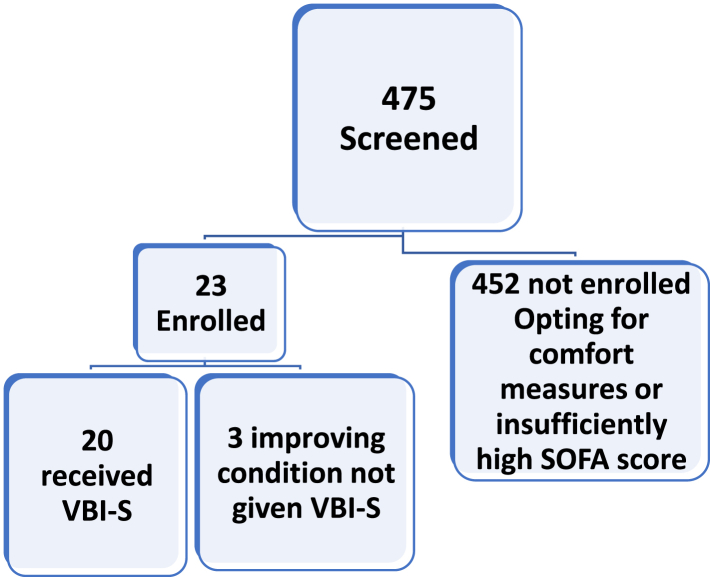
**Participant flow diagram.** Of the 475 patients reviewed for participation in the study only twenty-three were enrolled. Three of the patients did not receive VBI-S because their vasopressor dose was being weaned down.

Twenty patients were enrolled into the study. Patients were recruited between 2/17/2020 and 1/3/2023. Four hundred and seventy-five patients were reviewed for eligibility. Consent was obtained from the legally authorized representative of twenty-three patients and were screened. Three of these patients were not given VBI-S because their condition was improving as evidenced by the rapid decrease in their vasopressor dose. The purpose of this study was to determine efficacy of VBI-S in patients that were most likely to die. Therefore initially, only patients with SOFA scores of 15 or higher were enrolled. However, because of the severity of illness and the improbability of an acceptable quality of life many families opted for comfort measures only. Consequently, the rate of enrollment was very low. The slow enrollment was exacerbated by the COVID pandemic. On August 19, 2020 the FDA approved a lower minimum SOFA score of 12. But this failed to increase the rate of enrollment. On December 4, 2021 the FDA approved a reduction of the minimum SOFA score to 5. Supplementary Table S2 shows the protocol amendments for these changes. The lowering of the required SOFA score coupled with the abatement of the pandemic led to an acceleration of enrollment and its completion on 1/3/23. In spite of the lowering of the minimum SOFA score to 5 the initial SOFA scores of the enrolled patients was high. The initial SOFA score ± SD of these 20 patients prior to receiving VBI-S was 14.00 ± 2.92, with a median of 15, median IQR = 4.5 and a range of 8–18. The participant flow diagram is shown below in Fig. 1. Table 1 shows the patient demographics, diagnoses, body mass index (BMI) and initial SOFA scores of each patient.

**Table 1:** Demographics, diagnoses, body mass index (BMI) and initial pre-VBI-S SOFA score of individual patients.

Patient/gender	Age (years)	Diagnosis	BMI kg/m^2^	SOFA score
01-001-001/F	65	Sepsis of unknown origin Cardiac tamponade Cardiac arrest × 2	39.8	17
01-012-001/M	39	End stage respiratory failure due to COVID pneumonia/Bacterial pneumonia Acidosis (pH = 7.159) Hypercarbia (pCO_2_ = 89.2)	42.2	17
01-012-002/M	47	Infected pancreatic pseudocyst	33.2	16
01-012-003/M	77	Multiple gastric perforations Peritonitis Respiratory and metabolic acidosis (pH = 6.965)	24.1	18
01-012-004/F	66	Perforated sigmoid bowel Extensive fecal peritonitis Stage IVB Ovarian Carcinoma	22.7	17
01-012-005/M	81	Small Bowel perforation Adrenal cancer Colon cancer	33.1	17
01-012-006/M	61	Perforated sigmoid colon secondary to diverticulitis Necrotic gallbladder Metabolic acidosis (pH = 7.263)	20.2	12
01-012-007/M	56	Necrotizing pneumonia, empyema ARDS Neurogenic shock due to unstable fracture C4–C6 Metabolic acidosis (pH = 7.253)	20.4	12
01-012-008/M	53	Necrotizing pneumonia Metastatic pancreatic neuroendocrine carcinoma, hepatic cirrhosis, aspiration pneumonia, Pancytopenia due to chemotherapy, adrenal insufficiency, sick sinus syndrome Chronic pancreatitis	24.8	13
01-012-009/F	66	Necrotizing perineal fasciitis Metabolic acidosis (pH = 7.289)	36.8	9
01-006-002/M	78	Bacterial pneumonia, cardiomyopathy, hyperlipidemia	41.3	13
01-006-003/F	76	Urosepsis, acute encephalopathy, septic cardiomyopathy, COVID + Cerebrovascular accident	35.5	8^a^
01-006-004/F	68	Bacterial pneumonia, hyperlipidemia	26.7	15
01-006-005/M	76	Pyelonephritis, septic cardiomyopathy, Myocardial Infarction	28.3	16
02-011-001/F	76	Urosepsis, metastatic GI endocrine tumor, pneumonia, kidney transplant, multisystem organ failure	30.8	16
02-011-002/F	70	Infected sacral decubitus ulcer, hyperlipidemia, end stage renal disease	32.1	12
02-011-003/F	74	Pneumonia due to COVID-19, Diabetes (hyperosmolar hyperglycemic state), rhabdomyolysis Metabolic acidosis (pH = 7.22)	31.9	15
02-011-005/M	62	Sepsis unknown origin, hypothermia, metabolic encephalopathy, pancreatitis Metabolic acidosis (pH = 7.0)	25.3	15
01-003-001/M	54	Perforation of gastric ulcer with hemorrhage, alcohol induced pancreatitis	21.2	12
01-004-002/M	75	Urosepsis Metabolic acidosis (pH = 7.245)	26.4	10
Mean ± SD (95% CI)	66.00 ± 11.47 (60.97, 71.03)		29.84 ± 6.91 (26.81, 32.87)	14.00 ± 2.92 (12.72, 15.28)

Patient age, diagnoses, BMI (body mass index) and baseline SOFA score prior to receiving VBI-S.

All numerical values are mean ± SD and 95% confidence intervals.

F = female, M = male.

a SOFA score does not reflect the severity of this patient’s illness. She was found to have severe septic cardiomyopathy early in her evaluation. Because of the patient’s poor quality of life prior to admission to the ICU the family decided that the patient should be given vasopressors and VBI-S and that no other measures should be deployed beyond attempting to elevate her blood pressure. Hence, she was not intubated and other measures such as mechanical enhancement of cardiac output or ECMO were not deployed. Nonetheless, this patient was included in the calculation of the mean SOFA score.

### Efficacy

The primary endpoint of this study was to increase mean blood pressure by at least 10 mmHg with infusion of VBI-S. All patients were on vasopressors at the time that VBI-S was administered. Each of the twenty patients met the primary endpoint. The baseline MAP prior to VBI-S infusion ± SD was 64.5 ± 4.27 with a range of 52.0–70.0. The median was 65.0 and in quartile 1 it was 62.0 and quartile 3 it was 68.0. The MAP taken immediately after the VBI-S infusion was 77.5 ± 4.73 mmHg with a range of 73.0 mmHg–80.0 mmHg. The median was 78.0 and in quartile 1 it was 73.5 mmHg and quartile 3 it was 80.0 mmHg. This difference between the means was statistically significant with p value = 0.0087 and 95% confidence interval (CI) for the increase in MAP of 10.86–15.15 mmHg.

The mean time ± SD needed to achieve an increase in MAP of ≥10 mmHg was 90.35 ± 101.40 min. The median time was 58 min with a range of 15–378 min. During this time no other medication was given or action taken that could have raised blood pressure.

The mean volume of VBI-S ± SD needed to achieve the primary endpoint was 561.0 ± 372.3 ml. The total mean volume of VBI-S ± SD given during the 48-h treatment period was 905.1 ± 352.00 ml.

Table 2 shows the MAP before and after VBI-S, the time required to meet the primary endpoint of an increase in MAP of ≥10 mmHg, the volume of VBI-S required to meet the primary endpoint and the total volume of VBI-S administered during the 48 h treatment period.

**Table 2:** Change in MAP before and after VBI-S with time and volume of VBI-S needed to reach primary endpoint and total VBI-S infused over 48 hours.

Patient	MAP (mmHg) before VBI-S	MAP (mmHg) after VBI-S	Time (minutes) required to meet primary endpoint	Volume (ml) of VBI-S required to reach primary endpoint	Total VBI-S volume (ml) given within 48 h
01-001-001	68	80	15	300	765
01-012-001	68	78	28	457	914
01-012-002	62	72	100	870	870
01-012-003	60	71	28	300	1500
01-012-004	65	75	326	1300	1300
01-012-005	67	77	20	192	592
01-012-006	68	79	22	300	617
01-012-007	65	77	26	341	890
01-012-008	68	78	144	1118	1118
01-012-009	69	79	135	800	800
01-006-002	62	73	25	300	700
01-006-003	63	80	178	1459	1459
01-006-004	61	72	64	416	416
01-006-005	67	82	61	673	880
02-011-001	60	70	378	600	1300
02-011-002	68	79	95	560	580
02-011-003	70	81	24	350	350
02-011-005	52	74	60	400	1500
01-003-001	65	84	22	383	630
01-004-002	63	89	56	100	920
Mean ± SD (95% CI)	64.5 ± 4.3 Median = 65 Q_1_ = 62 Q_3_ = 68 (62.62, 66.38)	77.5 ± 4.7^a^ Median = 78 Q_1_ = 73.4 Q_3_ = 80.0 (75.44, 79.56)	90 ± 101.40 Median = 58 Q_1_ = 24.5 Q_3_ = 117.5 (45.56, 134.44)	561.0 ± 372.3 Median = 408 Q_1_ = 300 Q_3_ = 736.5 (397.83, 724.17)	905.1 ± 352.2 Median = 875 Q_1_ = 623.5 Q_3_ = 1209 (750.74, 1059.46)

Change in MAP before and after VBI-S, time of VBI-S infusion needed to achieve the primary endpoint of an increase in MAP ≥10 mmHg, the volume of fluid needed to reach the endpoint and the total volume of VBI-S infused over the 48 hour treatment period. All numerical values are mean ± SD, median, Q_1,_ Q_3_ and 95% confidence interval. p = 0.0087 for the difference between MAP before and after VBI-S infusion.

a Statistically significant.

The secondary endpoint was to achieve a decrease in the dose of vasopressors. Prior to receiving VBI-S patients were treated with 1–4 vasopressors. All vasopressor doses were converted to norepinephrine equivalents using the conversion factors found in Brand et al.^55^ The conversion was norepinephrine μg/minute = epinephrine μg/min = 0.1 × phenylephrine μg/minute = Dopamine μg/kg/minute × 0.5 = vasopressin units/minute × 60 × 5.6. All patients were given norepinephrine tartrate with dosing expressed as norepinephrine base. The baseline dose of norepinephrine equivalents ± SD was 39.63 ± 52.1. Within 48 h the vasopressor dose was weaned down to 16.0 ± 39.9 (p = 0.0011). In three of the 20 patients weaning was not initiated within 1 h after infusion of VBI-S. Therefore, the decrease in vasopressor dose could not be attributed to VBI-S with certainty. These patients were not included in the data analysis. Table 3 shows the lowering of vasopressor dose after VBI-S elevated MAP by ≥10 mmHg.

**Table 3:** Vasopressor dose as norepinephrine equivalents before and after VBI-S with time required to wean vasopressor to lowest point within the 48 hour treatment period.

Patient	Norepinephrine equivalents μg/min before VBI-S	Lowest Norepinephrine equivalents μg/min within 48 h after starting VBI-S	Time to wean to lowest vasopressor dose after VBI-S infusion (minutes)
00-000-LUT	45.4	23.4	675
01-012-001	3.5	0	23
01-012-002	64.4	14.5	556
01-012-003	219.6	163.4	646
01-012-004	22.1	0	1440
01-012-005	12.5	0	1101
01-012-006	17.5	0	676
01-012-007	31.7	0	1628
01-012-008	38.8	0	2085
01-012-009	ND	ND	ND
01-006-002	4.0	0	1030
01-006-003	2.0	44.1	ND
01-006-004	42.1	0	2610
01-006-005	46.1	0	1901
02-011-001	88.2	20.2	881
02-011-002	ND	ND	ND
02-011-003	12	0	458
02-011-005	ND	ND	ND
01-003-001	21.3	5.98	2880
01-004-002	3.0	0	256
Mean ± SD 95% CI	39.7 ± 52.1 Median = 22.1 Q_1_ = 12 Q_3_ = 45.4 (14.93, 64.47)	16.0 ± 39.9^a^ Median = 0 Q_1_ = 0 Q_3_ = 14.5 (0, 34.97)	1117.9 ± 838.94 Median = 955.5 Q_1_ = 623.5 Q_3_ = 1696.3 (706.82, 1528.98)

Change in norepinephrine equivalents after the infusion VBI-S and the time required to reduce vasopressor dose to the lowest level within the 48 hour treatment period. All numerical values are mean ± SD, median, Q_1,_ Q_3_ and 95% confidence interval.

ND = weaning not done.

p = 0.0011 for the difference between the baseline Levophed equivalents before and after VBI-S. The time required to wean down to the lowest dose of vasopressor of the 48-h treatment period is shown.

a Statistically significant.

Eleven of the 20 patients were weaned to zero vasopressor within 48 h. Weaning required a mean time of 1177.9 ± 838.9 min and a median of 1064.4 min with a range of 23–2610 min to reach the lowest level achieved within the 48-h treatment period.

In addition to the anticipated increase in blood pressure and reduction of vasopressor dose, we observed improvement in oxygenation at 24 and 48 h as evidenced by an increase in the ratio of the partial pressure of oxygen in the arterial blood over the fraction of inspired oxygen. We also observed improvement in renal function as a reduction in creatinine at 48 h. The reduction of the AST/ALT ratio at 24 h suggests that VBI-S reduced the progression of liver dysfunction. Coagulation variables also normalized. These improvements in the cardiovascular, pulmonary, renal, hepatic and coagulation systems occurred without injury to the heart as shown by absence of a significant change in troponin or the EKG. Similarly, there was no finding that would suggest damage to the hepatobiliary or pancreatic systems after VBI-S infusion. There was a significant decrease in lipase at 48 h. Normalization of coagulation indices and the decrease in procalcitonin are consistent with a marked reduction in the inflammatory response. Within 24 h after VBI-S there was a significant decrease in lactate suggesting improved tissue perfusion. However, within 48 h after VBI-S there was no difference. The SOFA score of the patients was significantly improved at 24 h after receiving VBI-S. This improvement was maintained at 48 h. Table 4 shows the changes in the laboratory values and SOFA scores over 24 and 48 h.

**Table 4:** Effect of VBI-S on organ systems and SOFA score.

	Mean ± SD at baseline before VBI-S and (number of patients)	Mean ± SD at 24 ± 6 h after VBI-S (number of patients and p value) 95% CI	Mean ± SD at 24–48 ± 6 after VBI-S (number of patients and p value) 95% CI
**Respiratory system**			
PaO_2_/FiO_2_ ratio/100 (mmHg)	2.00 ± 0.84 (17)	2.6 ± 0.68 (12, 0.0024)^a^ (2.26, 3.03)	2.5 ± 0.76 (12, 0.0210)^a^ (2.07, 2.93)
PaO_2_ (mmHg)	102.6 ± 30.51 (18)	131.0 ± 60.56 (12, 0.0425)^a^ (96.74, 165.3)	127.8 ± 57.48 (12, 0.2036) (95.28, 160.31)
PaCO_2_ (mmHg)	43.6 ± 9.85 (20)	43.1 ± 8.05 (15, 0.7958) (39.03, 47.17)	37.5 ± 8.53 (14, 0.0197)^a^ (33.03, 41.97)
FiO_2_ (%)	56.2 ± 22.38 (19)	49.7 ± 17.33 (14, 0.6875) (39.70, 59.70)	50.9 ± 19.21 (15, 0.7168) (40.29, 61.51)
pH	7.1 ± 0.14 (17)	7.3 ± 0.08 (15, 0.5114) (7.26, 7.34)	7.4 ± 0.14 (14, 0.2734) (7.32, 7.48)
HCO_3_ (blood gas) (mmol/L)	21.6 ± 5.24	23.9 ± 5.20 (14, 0.2223) (20.90, 26.90)	22.3 ± 7.63 (14, 0.6233) (17.90, 26.71)
Base excess (mEq/L)	−3.9 ± 7.33 (18)	−2.1 ± 5.70 (13, 0.3013) (−1.35, 5.55)	−4.7 ± 7.81 (12, 0.9170) (−0.26, 9.66)
**Renal system**			
Creatinine (mg/dL)	2.4 ± 1.23 (20)	2.2 ± 1.26 (16, 0.2979) (1.53, 2.87)	1.7 ± 0.90 (16, 0.0076)^a^ (1.22, 2.18)
BUN (mg/dL)	39.3 ± 18.40 (20)	45.1 ± 21.28 (15, 0.2554) (33.34, 56.86)	38.5 ± 21.83 (15, 0.6146) (26.44, 50.56)
**Hepatobiliary pancreatic system**			
Aspartate amino transferase (AST) (U/L)	732.0 ± 2216.85 (20)	473.1 ± 1332.18 (17, 0.4514) (−211.87, 1158.07)	362.3 ± 700.77 (15, 0.9453) (−24.91, 749.51)
Alanine amino transferase (ALT) (U/L)	297.5 ± 701.32 (20)	270.3 ± 799.06 (17, 0.0561) (−140.56, 681.16)	251.9 ± 626.04 (15, 0.2346) (−94.02, 597.82)
AST/ALT	2.1 ± 0.89 (16)	1.7 ± 0.95 (13, 0.0105)^a^ (1.13, 2.27)	2.1 ± 1.48 (12, 0.1763) (1.16, 3.04)
Alkaline phosphatase (IU/L)	150.8 ± 202.06 (18)	174.5 ± 223.50 (13, 0.5693) (39.37, 309.63)	200.1 ± 210.93 (12, 0.3086) (66.14, 334.06)
Total bilirubin (mg/dL)	1.8 ± 1.51 (20)	1.7 ± 1.20 (14, 0.2678) (−0.7, 4.10)	1.9 ± 1.12 (12, 0.7646) (1.28, 2.52)
Direct bilirubin (mg/dL)	1.1 ± 1.06 (17)	0.9 ± 0.89 (11, 0.0508) (0.30, 1.50)	1.2 ± 0.99 (10, 0.8125) (0.49, 1.91)
Indirect bilirubin (mg/dL)	1.0 ± 0.88 (13)	1.2 ± 0.85 (9, 0.6250) (0.55, 1.85)	0.8 ± 0.43 (7, 0.3750) (0.40, 1.20)
Lipase (U/L)	55.1 ± 122.27 (19)	34.3 ± 26.72 (13, 0.0962) (18.14, 50.46)	51.6 ± 62.08 (9, 0.0234)^a^ (3.80, 99.40)
Amylase (U/L)	166.1 ± 301.54 (18)	47.6 ± 35.86 (12, 0.5391) (24.83, 70.37)	40.6 ± 32.75 (9, 0.9102) (15.38, 65.82)
Triglycerides (mg/dL)	241.8 ± 205.98 (18)	764.3 ± 725.07 (15, 0.0134)^a^ (363.67, 1164.93)	417.1 ± 584.60 (12, 0.0444)^a^ (45.83, 788.37)
**Coagulation system**			
International normalized ratio	1.9 ± 0.69 (18)	1.5 ± 0.83 (14, 0.02)^a^ (1.06, 1.93)	1.2 ± 0.28 (10, 0.004)^a^ (1.03, 1.37)
Prothrombin time (seconds)	21.6 ± 7.27 (18)	17.4 ± 9.86 (14, 0.013)^a^ (12.24, 22.56)	14.3 ± 3.22 10, 0.004^a^ (12.30, 16.30)
Partial thromboplastin time (seconds)	48.3 ± 16.78 (16)	42.9 ± 17.11 (14, 0.37) (33.94, 51.86)	42.9 ± 27.54 (11, 0.43) (26.65, 59.15)
Platelet count (cells/ml)	146.6 ± 81.74 (19)	140.4 ± 78.85 (16, 0.02)^a^ (101.80, 179.0)	110.9 ± 74.23 (15, 0.0006)^a^ (73.3, 148.50)
**Inflammatory response**			
Procalcitonin (ng/ml)	100.3 ± 180.88 (15)	62.1 ± 118.09 (13, 0.0007)^a^ (−2.09, 126.3)	57.4 ± 97.21 (9, 0.004)^a^ (−6.11, 120.9)
White blood cell count (cells/μl)	23.8 ± 16.72 (20)	24.5 ± 14.31 (16, 0.4887) (17.49, 31.51)	27.4 ± 18.94 (15, 0.9515) (17.82, 36.98)
**Cardiovascular system/Tissue perfusion/Red cells**			
Troponin (ng/ml)	89.1 ± 217.61 (18)	44.0 ± 50.84 (15, 0.90) (18.27, 69.73)	27.3 ± 38.57 (11, 0.38) (7.78, 46.82)
Lactic acid (mmol/L)	3.8 ± 3.26 (20)	2.54 ± 2.63 (15, 0.03)^a^ (1.21, 3.87)	4.8 ± 5.55 (13, >1.00) (1.78, 7.82)
Hematocrit (%)	30.8 ± 5.87 (20)	30.0 ± 5.82 (16, 0.08) (27.15, 32.85)	29.4 ± 5.85 (15, 0.03)^a^ (26.44, 32.36)
Hemoglobin (grams/L)	9.9 ± 1.98 (19)	10.1 ± 2.05 (16, 0.58) (9.10, 11.1)	9.6 ± 1.96 (15, 0.018)^a^ (8.61, 10.59)
**Electrolytes**	137.5 ± 4.44 (20)	137.2 ± 5.44 (15, 0.28) (134.40, 140.00)	140.5 ± 3.96 (15, 0.01)^a^ (138.5, 142.5)
Potassium (mEq/L)	4.6 ± 0.65 (19)	4.4 ± 0.97 (14, 0.53) (3.89, 4.91)	4.0 ± 0.52 (13, 0.006)^a^ (3.72, 4.28)
Chloride (mEq/L)	98.4 ± 25.35 (17)	102.9 ± 6.20 (14, 0.64) (99.65, 106.1)	93.4 ± 33.47 (14, 0.43) (75.87, 110.90)
HCO_3_ (Blood) (mmol/L)	21.2 ± 5.58 (16)	22.9 ± 5.08 (10, 0.94) (19.75, 26.05)	31.7 ± 26.48 (9, 0.78) (14.40, 49.00)
Glucose (mmol/L)	161.3 ± 72.83 (20)	171.5 ± 83.49 (15, 0.97) (129.20, 213.80)	131.6 ± 62.13 (15, 0.12) (100.20, 163.00)
**SOFA score**	14.0 ± 2.92 (20)	11.4 ± 3.88 (13, 0.02)^a^ (9.29, 13.51)	11.4 ± 3.58 (12, 0.008)^a^ (9.37, 13.43)

Multiple systemic parameters as mean ± SD, the number of patients, the p value compared to baseline and the 95% confidence interval.

Baseline values are within 6 h prior to administration of VBI-S.

a Statistically significant.

### Safety

There were no severe adverse events due to the investigational product, VBI-S. No abnormalities in vital signs or EKG were attributed to the investigational product. No abnormalities in the pulse, electrocardiogram, or temperature were attributed to VBI-S. No new infections after VBI-S were observed. By 48 h the hematocrit and hemoglobin were decreased. These decreases were indistinguishable from the expected decrease previously reported in critically ill patients.^56^ Similarly, there was a decrease in the platelet count that also was within the expected range of patients in septic shock.^57^ This decrease in platelet count resolved within one week after VBI-S. There were numerous treatment emergent adverse events that occurred after VBI-S was infused in these severely ill patients as shown in Supplementary Table S3. The only adverse event that was definitely related to VBI-S was hyperlipidemia (defined as a triglyceride level of greater than 150 mg/dL). 11/18 (57%) of the patients had hyperlipidemia at baseline prior to VBI-S. There was a statistically significant difference in the triglyceride level at 24 and 48 h after VBI-S with 12/15 (80%) and 9/12 (75%) patients having hyperlipidemia, respectively (Table 4). One week after receiving VBI-S there was no significant difference between the triglyceride level and baseline (data not shown).

No adverse effect from the decrease in hematocrit and hemoglobin, decreased platelet count or hyperlipidemia was observed. The therapeutic objective was to create a hydrophobic region within the intravascular space into which excessive lipophilic nitric oxide enters. This was done by drastically increasing the volume of lipid in the bloodstream within an acute timeframe.

## Discussion

The efficacy of VBI-S in elevating the blood pressure and reducing vasopressor dose in this challenging population demonstrates that VBI-S could improve the possibility of survival for patients in the advanced stages of septic shock. The salutary effects on multiple failing organ systems, and reduction of inflammation further support the probability that VBI-S enhances survival of these patients. Given its beneficial effect on multiple systems namely the cardiovascular, pulmonary, and renal systems, VBI-S could be a treatment for the multiple organ dysfunction syndrome.

No severe adverse effects were observed. The hyperlipidemia was transient and did not result in any adverse effects. The decrease in platelets, haematocrit and haemoglobin were consistent with expected effects in septic patients. Ten of the 20 patients were discharged from the hospital. Four other patients had a decrease in mean SOFA score ± SD from 16.00 ± 0.82 to 9.50 ± 3.42 in 24 h. But their families decided it was in the patient’s best interest not to continue with full-scale intervention since the long-term prospect of an acceptable quality of life after discharge from the ICU was low. The remaining 6 patients died before 24 h after giving VBI-S.

Our results are consistent with the reduction of NO bioavailability in regions where its concentration is excessive leading to an increase in blood pressure and a reduction in vasopressor dose. They are also consistent with improvement of organ function by the delivery of NO to regions of the microcirculation in which NO concentration is too low, by off-loading from VBI-S. The concentration of the highly toxic free radical peroxynitrite, could be diminished by the reduction of NO bioavailability and consequently also contribute to a reduction in organ injury. The oxygen carrying capability of VBI-S could also play a role by delivering oxygen to regions of the microcirculation that had insufficient blood flow until being opened by the delivery of NO via VBI-S. The small size of the nanoparticles could facilitate oxygen delivery. There are additional interactions that could play a role that will be investigated such as interactions between phospholipid nanoparticles and other endogenously produced lipophilic gases such as carbon monoxide^58^ and hydrogen sulphide.^59^ Non-responsiveness of hypotension to standard fluids that occurs in medical conditions other than septic shock could also possibly be treated with VBI-S.

This was a repeated measures design in which treatment of hypotension with standard fluids such as Ringer’s lactate, normal saline or 5% albumin or 25% albumin failed to elevate blood pressure to at least a MAP of 65 mmHg. This failure was followed by administration of vasopressors. While the patient was receiving vasopressors the test fluid, VBI-S, was given. In this study the baseline data taken prior to VBI-S are compared to data taken after VBI-S infusion. This design is prone to error because the order of treatments could make a difference. In this case the order cannot be reversed because standard fluids with a goal of elevating MAP by 10 mmHg are contraindicated after they have failed. Because of the need to individualize fluids for this severely ill patient population no specific volume was required before it was determined that the patients had failed fluid therapy for the reversal of hypotension. This determination was made by each patient’s physician. Because VBI-S was not given immediately after failure of conventional fluids it is possible that at the time the patient received VBI-S there were some patients who had a combination of insufficient intravascular volume (absolute hypovolemia) and vasodilation (relative hypovolemia). Currently undetected adverse effects may be revealed with administration of VBI-S to more patients. Although multiple correlates of improved survival were observed in this study a definitive conclusion about the survival benefit of VBI-S cannot be made without a randomized controlled study.

## Contributors

CS invented the technology, designed the study, was the overall PI of the study and wrote the manuscript of this paper. MM, HK, KR, SB, LD-C, JC, DH and AM were principal investigators at their respective sites and screened patients for enrollment in the study. MK coordinated the execution of the study. HD contributed to study design. JR made the measurements of NO and oxygen uptake of the phospholipid nanoparticles. PA assisted with data analysis and with writing the manuscript. NH made preliminary clinical observations on the efficacy of VBI-S. BM performed data analysis and assisted with the writing of the manuscript. KT contributed to study design and assisted with the writing of the manuscript. MM and PA had access to the data and verified it.

## Data sharing statement

Upon publication, and after de-identification the data supporting the results of this article will be made available to researchers who provide a methodologically sound proposal. The de-identification will be carried out in compliance with applicable privacy laws, data protection, and requirements of anonymization and consent. Requests for access to the data should be directed to the corresponding author.

## Declaration of interests

Ten US patents and 35 patents in international jurisdictions have been issued to CS for the phospholipid nanoparticle technology. Two patents are pending. One patent application is for the treatment of multiple organ dysfunction syndrome. The other is for the use of phospholipid nanoparticles to reduce inflammation. CS is the principal investigator of the grant from the Naval Medical Research Command. CS is President of Vivacelle Bio, Inc., the company that is the sponsor of the trial and its board chairman. He is also a Vivacelle Bio shareholder. Support for attending scientific meetings is provided to CS by Vivacelle Bio and the UMKC School of Medicine. MK is a contractor for the clinical trial and was paid for his contract work. He is the Senior Vice-President of Vivacelle Bio, Inc., and a Vivacelle Bio Shareholder. HD is CEO of Vivacelle Bio and board member of Vivacelle Bio. He is also a Vivacelle Bio shareholder. Travel expenses are provided by Vivacelle Bio and 2Flo Ventures, an investor in Vivacelle Bio. JR is a member of Vivacelle Bio’s Scientific Advisory Board. Vivacelle Bio gave JR a nitric oxide detector that was used in the manuscript. NH is on the advisory Board of Vivacelle Bio and a Vivacelle Bio shareholder. KT is Chief Medical Officer of Vivacelle Bio and a shareholder of Vivacelle Bio and of Johnson and Johnson. No other authors have a conflict of interest.
