# Supplementary material for: Efficacy and safety of phospholipid nanoparticles (VBI-S) in reversing intractable hypotension in patients with septic shock: a multicentre, open-label, repeated measures, phase 2a clinical pilot trial
Source: eClinicalMedicine. 2024 Jan 29;68:102430. doi: 10.1016/j.eclinm.2024.102430 (PMC10965406; doi:10.1016/j.eclinm.2024.102430)
Supplement: Supplementary Materials [file mmc1.docx]

**Supplementary Figure 1**

**Supplementary Table 1**

| **Nitric Oxide-Water** | **Nitric Oxide-VBI-S** | **Oxygen- Water** | **Oxygen- VBI-S** |
| --- | --- | --- | --- |
| 173.6 | 249.8 | 4619 | 6953 |
| 125.1 | 240.9 | 4276 | 6022 |
| 126.1 | 273.1 | 4250 | 6433 |
| 97.6 | 293.4 | 5025 | 7368 |
|  |  | 5123 | 7072 |
| Mean=130.60 | Mean=264.30 | Mean = 4658.60 | Mean =6769.60 |
| SD=31.56 | SD= 23.68 | SD=407.68 | SD=240.35 |
|  | p=0.0005 |  | p=0.0001 |

**Supplementary Figure 2**

**Flow Chart of Patient Management**

**All Hypotensive Septic Patients with MAP < 65 mmHg Not Responsive to Fluids on Vasopressors and Condition Not Improving**

**VBI-S Titrated to Goal of Increased MAP by ≥ 10 mmHg**

**Wean Vasopressors + Supplemental VBI-S to goal MAP of 60-65**

**During 48-hour treatment period**

**Weekly Follow up to 28 days, Discharge or Death**

**More VBI-S as decided by the site physician**

**Supplementary Figure 3 Q-Q Plot**

**Supplementary Table 2**

| **Protocol Version** | **Date** | **Changes Made** | **FDA Approval** | **IRB Approval** |
| --- | --- | --- | --- | --- |
| 1.0 | 27 May 2019 | Original protocol | 5 July 2019 | 31 December 2019 |
| 1.1 | 20 July 2020 | Revise Inclusion criteria, SOFA score ≥15 to SOFA score ≥12 | 19 August  2020 | 18 Aug 2020 |
| 2.0 | 6 April 2021 | Amendments:  Changed to multi-site trial  Allowed inclusion of patients on Covid treatments | 11 May  2021 | 16 April 2021 |
| 3.0 | 5 Nov 2021 | Revise Inclusion criteria, SOFA score ≥12 to SOFA score ≥5 | 4 December 2021 | 16 November 2021 |

**Supplementary Table 3**

**Treatment Emergent Adverse Events (TEAE) by System Organ Class**

| **System Organ Class** | **Overall**  **(N=20 patients)**  **Subject Events**  **n % E** |
| --- | --- |
| **Total TEAE** | **14 (70.0%) 36** |
|  |  |
| **CARDIAC DISORDERS** |  |
| **Atrial Fibrillation** |  |
| **Cardiac Arrest** | **3 (15.0%) 3** |
| **Tachycardia** | **1 (5.0%) 1** |
| **Ventricular Tachycardia** | **1 (5.0%) 1** |
| **GASTROINTESTINAL DISORDERS** |  |
| **Dysphagia** | **1 (5.0%) 1** |
| **Pancreatitis** | **1 (5.0%) 1** |
| **Splenic artery aneurysm** | **1 (5.0%) 1** |
| **GENERAL DISORDERS and GENERAL SITE CONDITIONS** |  |
| **Pyrexia** | **1 (5.0%) 1** |
| **HEPATOBILIARY DISORDERS** |  |
| **Ischemic Hepatitis** | **1 (5.0%) 1** |
| **INFECTIONS and INFESTATIONS** | **7 (35.0%) 8** |
| **HERPES VIRUS INFECTION** | **1 (5.0%) 1** |
|  |  |

**n = number of patients. E = number of events**

VBI-S for the Treatment of Hypotension in Hypovolemic Septic Shock Patients

**Protocol Number: VBI-S-01 Version: 3.0**

**Date: 5 Nov 2021**

# SYNOPSIS

| **Name of Sponsor:**  Vivacelle Bio, Inc. | |
| --- | --- |
| **Name of Investigational Product:**  VBI-S | |
| **Title of the Study:**  VBI-S for the treatment of Hypotension in Hypovolemic Septic Shock Patients | |
| **Protocol Number: VBI-S-01** | **Indication: Reversal of Hypotension in Hypovolemic Septic Shock Patients** |
| **Study center(s): Multi-center** | |
| **Principal Investigator: Cuthbert O. Simpkins, MD, FACS** | |
| **No. of Patients: 20** | **Phase of development: IIa, POC** |
| **Objectives Primary:**  The primary objective of this study is to evaluate the safety and efficacy of VBI-S in elevating the blood pressure in septic shock patients with absolute or relative hypovolemia.  **Secondary:**  The secondary objective of this study is to determine the use of pressor drugs after infusion of VBI-S.  **Endpoints Primary:**  The primary endpoint of this study is the proportion of patients in whom mean blood pressure increased by at least 10 mmHg with a mean blood pressure target of 60 – 65 mmHg.  **Secondary:**  The secondary endpoint of this study is the proportion of patients in whom the dose of pressor drugs could be decreased after infusion of VBI-S to maintain a mean arterial pressure of 60-65 mmHg. | |

| **Exploratory:**  The tertiary and exploratory endpoints of this study are:   1. Proportion of patients that need the change in the dose and type of vasoactive drugs post administration of VBI-S infusion 2. 28-day mortality post-administration of VBI-S infusion 3. Duration of ICU stay 4. Duration of hospital stay 5. Proportion of patients with new infections after receiving VBI-S 6. Change in volume status as indicated by the CVP, ultrasound assessment of the inferior vena cava or internal jugular vein, or changes in stroke volume index or cardiac output 7. Change in Sequential Organ Failure Assessment Score 8. Change in Arterial Oxygen saturation and partial pressure of oxygen and carbon dioxide, base excess post‐infusion 9. Change in serum lactate 10. Change in platelet count 11. Change in Prothrombin Time (PT) and INR 12. Change in Partial Thromboplastin Time (PTT) 13. Change in serum sodium, potassium, chloride, HCO3, CBC, Blood Urea Nitrogen (BUN) creatinine, liver function tests, amylase, lipase, troponin and lactate. 14. Change in plasma triglycerides |
| --- |
| **Study Design**: The aim of this Phase IIa a proof of concept, open label study to evaluate the safety and efficacy of VBI-S in elevating the blood pressure of septic shock patients with absolute or relative hypovolemia and who have a Sequential Organ Failure Assessment (SOFA) score of  ≥ 5. Blood pressure will be measured by a catheter in the arterial system or if an arterial line is unavailable by blood pressure cuff.  When a patient that meets the study inclusion criteria with no exclusion criteria, and consent to be part of the study has been obtained, an arterial line will be placed if it is not already in place. Baseline blood pressure will be recorded for 15 minutes to ensure that the measurements are stable. After the 15-minute observation period, VBI-S will be given in sequentially increasing volumes at a rate of 999 ml/hour to a maximum of 1500 ml of VBI-S with a goal to have an increase in the mean blood pressure of at least 10 mmHg or more. Repeat boluses will be given as needed to maintain an elevation of blood pressure of 10 mmHg or more and a MAP of 60-70 for a 48-hour trial period.  **Number of patients (planned):** 20 patients |
| **Inclusion criteria:**   1. Male or female at least 18 years of age 2. Evidence of bacterial infection demonstrated by positive blood culture and/or a known source of infection or an elevated procalcitonin of ≥ 2 ng/ml. 3. Patient has a mean blood pressure < 65 mmHg that is unresponsive to fluids currently   available on the market. |

| 1. Sequential Organ Failure Assessment (SOFA) score ≥ 5. 2. Sepsis diagnosis: The presence of infection which can be proven or suspected by 2 or more of the following criteria:    - Lactate > 2 mmol/L    - Mottled skin    - Decreased capillary refill of nail beds or skin    - Fever > 38.3˚C, or 101˚F    - Hypothermia < 36˚C core temperature (<96.8˚F)    - Heart rate > 90    - Tachypnea    - Change in mental status    - Significant edema or positive fluid balance (>20 mL/kg over 24 hours)    - Hyperglycemia (>140 mg/dL) in someone without diabetes    - White blood cell count >12,000 or less than 4,000, or with >10% "bands" (immature    - forms)    - Elevated C-reactive protein in serum (according to your lab's cutoffs)    - Elevated procalcitonin in serum (≥ 2 ng/ml)    - Arterial hypoxemia (PaO2/FiO2 < 300)    - Acute drop in urine output (<0.5 ml/kg/hr for at least 2 hours despite fluid resuscitation, or about 30 ml/hour for a 70 kg person)    - Creatinine increase > 0.5 mg/dL    - INR > 1.5 or aPTT > 60 seconds    - Absent bowel sounds (ileus)    - High bilirubin (total bilirubin > 4 mg/dL   **Exclusion Criteria:**   1. Patients with a ventricular assist device 2. Acute coronary syndrome 3. Pregnant 4. Bronchospasm 5. Mesenteric ischemia 6. Emergency surgery 7. Acute liver disease (Hepatitis B and C as examples) 8. Liver failure with a Model for End-Stage Liver Disease (MELD) score ≥ 19 9. Hematologic or coagulation disorders including thrombocytopenia (platelet count   <50,000) and associated with hemodynamically significant active bleeding.   1. Absolute neutrophil count of < 1000 mm^3^ 2. Current participation or participation in another experimental or device study within the last 30 days before the start of this study. Patient may be included if on other drugs for COVID-19 and or septic shock 3. Patients with a known allergy to soybeans or eggs 4. Patient is hypervolemic as assessed by CVP, ultrasound, Swan Ganz catheter, Flo-Trac, esophageal doppler, bioimpedance, ECHO, Partial carbon dioxide rebreathing (NICO), lithium dilution (LIDCO) or other method published in a peer reviewed journal |
| --- |

| **Investigational product, dosage, and mode of administration:**  **Investigational product**: VBI-S, is a compounded soybean oil emulsion comprised of as weight/volume 20% soybean oil, 2.25% glycerin and 1.2% egg lecithin with NaCl at 77 meq added on the day of use.  **Dosage**:  After the 15-minute observation period, VBI-S will be given in continuously increasing volumes at a rate of 999 ml/hour to a maximum of 1500 ml. Repeat boluses will be given as needed to maintain an elevation of blood pressure of 10 mmHg or more or as needed to maintain a mean blood pressure of 60-70 mmHg for a 48-hour trial period.  **Mode of administration**: Intravenous Infusion |
| --- |
| **Treatment Duration:**  The treatment will last up to 48 hours after the first administration by repeating doses of VBI-S as needed to maintain a mean blood pressure of 60-70 mmHg or an elevation of blood pressure of at least 10 mmHg. |
| **Sample Size Considerations:**  The sample size is based on a hypothesized rate of achievement of the primary and secondary endpoint efficacy end point of 80%.  **Randomization and blinding:**  This is an open label study, randomization and blinding are not applicable to this study. All patients entering this study will be treated with VBI-S.  **Interim Analysis:**  Once the first 10 patients complete the day 28 assessment, an interim analysis of the data will be performed, and presented to the Data Safety Monitoring Board (DSMB). Based on their assessment the DSMB will recommend whether to continue to trial as planned, modified, or discontinued.  **Data Safety Monitoring Board:**  A Data Safety Monitoring Board (DSMB) will review the study data. The DSMB will monitor the safety of the trial from the beginning. The DSMB will consist of at least three members (combination of physicians/critical care nurses/regulatory experts) who will review all individual physiological variables and the safety laboratory values, all adverse events (AE) including serious AEs as well as unrelated and all related deaths during the Treatment and Follow-Up Phases. All utilized vasopressors used and their doses in each patient and the patient’s vitals at the time will also be provided. All expedited safety reports will be provided in real time to the DSMB chair upon being reported to FDA.  A further description of the DSMB reporting requirements, meeting frequency etc. can be found |

| in the DSMB charter.  **Analysis Populations:**   - Safety Analysis Set (SAS): all patients who have received any amount of VBI-S. - Intent to treat (ITT) population: all patients who have received any amount of VBI-S   **Statistical methods:**  The statistical analysis will be performed using SAS^®^ version 9.4 or higher. Descriptive statistics with 95% confidence intervals will be used to summarize data according to treatment group. Change in blood pressure to a hypothetical null result using an unpaired two tailed Student’s t test. P< 0.05 will be considered significant. All secondary and exploratory endpoints will be analyzed if the primary endpoint is met. The Kaplan – Meier Estimator will be used to describe patient survival.  **Safety Evaluation:**  **Adverse Events:** Adverse events will be coded using the most recent version of Medical Dictionary for Regulatory Activities (MedDRA). Treatment-emergent adverse events **(**TEAEs) are defined as events with an onset on or after the first dose of study drug infusion. TEAEs will be summarized by system organ class (SOC) and preferred term (PT).  The following summaries will be presented:  Summary of TEAEs by SOC and PT  Summary of TEAEs by SOC, PT, number and severity of event Summary of TEAEs by SOC, PT, maximum study drug relationship  Summary of TEAEs leading to discontinuation of study drug by SOC and PT Summary of TEAEs leading to death by SOC and PT  Summary of TEAEs related to study drug by SOC and PT Summary of TEAEs related to study drug by SOC, PT, and severity; Summary of serious TEAEs by SOC and PT;  Summary of serious TEAEs related to study drug by SOC and PT.  Data collected from other safety evaluations will be summarized descriptively and/or listed according to the data type. |
| --- |

**TABLE OF CONTENTS**

[TITLE PAGE 1](#_bookmark0)

[INVESTIGATOR’S SIGNATURE PAGE 2](#_bookmark1)

[PROTOCOL APPROVAL PAGE 3](#_bookmark2)

[PROCEDURES IN CASE OF EMERGENCY 4](#_bookmark3)

[SYNOPSIS 5](#_bookmark5)

[LIST OF TABLES 13](#_bookmark6)

[LIST OF FIGURES 14](#_bookmark7)

[LIST OF ABBREVIATIONS AND DEFINITIONS OF TERMS 15](#_bookmark8)

[ADR15](#_bookmark10)

[Adverse Drug Reaction 15](#_bookmark11)

[Suspected Adverse Reaction 16](#_bookmark12)

1. [INTRODUCTION 17](#_bookmark13)


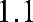
[Statement of Intent 17](#_bookmark14)


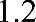
[Background 17](#_bookmark15)


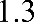
[Investigational Product: VBI-S 18](#_bookmark16)

- - 1. [Description 18](#_bookmark17)
    2. [Proposed Mode of Action and Treatment Rationale for this study 18](#_bookmark18)
    3. [Rationale for Dose Selection 19](#_bookmark19)
    4. [Previous Clinical Experience 19](#_bookmark20)
    5. [Risks and Benefits Assessment 21](#_bookmark23)
       1. [Potential Benefits 21](#_bookmark24)
       2. [Potential Risks 21](#_bookmark25)

1. [STUDY OBJECTIVES AND ENDPOINTS 22](#_bookmark26)


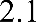
[Primary Objective(s) 22](#_bookmark27)


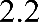
[Secondary Objective(s) 22](#_bookmark28)


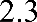
[Study Endpoints 22](#_bookmark29)

- - 1. [Primary Endpoints 22](#_bookmark30)
    2. [Secondary Endpoints 22](#_bookmark31)
    3. [Exploratory Endpoints 22](#_bookmark32)

1. [STUDY DESIGN 23](#_bookmark33)


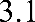
[Overall Study Design 23](#_bookmark34)


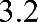
[Number of Patients 23](#_bookmark35)


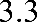
[Treatment Assignment 23](#_bookmark36)


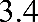
[Dose Adjustment Criteria 25](#_bookmark39)

- - 1. [Safety Criteria for Adjustment or Stopping Doses 25](#_bookmark40)


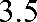
[Criteria for Study Termination 25](#_bookmark41)

- - 1. [Early discontinuation of this study 25](#_bookmark42)
    2. [Discontinuation of individual patient 25](#_bookmark43)


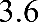
[Data Safety Monitoring Board (DSMB) 26](#_bookmark44)

1. [SELECTION AND WITHDRAWAL OF PATIENTS 27](#_bookmark45)


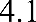
[Patient Inclusion Criteria 27](#_bookmark46)


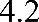
[Patient Exclusion Criteria 27](#_bookmark47)


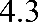
[Patient Withdrawal Criteria 28](#_bookmark48)

- - 1. [Data Collection from Withdrawn Patient 29](#_bookmark49)

1. [STUDY TREATMENT 30](#_bookmark50)


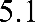
[Treatment Compliance 30](#_bookmark52)


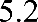
[Study Schedule 30](#_bookmark53)

- - 1. [Screening Phase 30](#_bookmark54)
    2. [Baseline Phase: 15- minute pre-infusion observation period 32](#_bookmark55)
    3. [Treatment Phase: Intensive Care Unit (ICU)-48- hour trial period. 32](#_bookmark56)
    4. [Follow-up Phase: Hospital Ward-28 days 32](#_bookmark57)

1. [STUDY DRUG MATERIALS AND MANAGEMENT 33](#_bookmark58)


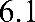
[Study Drug 33](#_bookmark59)

- - 1. [Description of Investigational Product 33](#_bookmark60)
    2. [Drug Substance 33](#_bookmark61)
    3. [Drug Product 33](#_bookmark62)
    4. [Drug Packaging and Labeling 33](#_bookmark63)
    5. [Drug Storage and Handling 34](#_bookmark64)


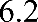
[Investigational Product Administration 34](#_bookmark65)


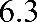
[Investigational Product Accountability 34](#_bookmark66)


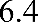
[Concomitant Medications and Contraindication 34](#_bookmark67)

- - 1. [Prohibited Medicine and Therapies 34](#_bookmark68)
    2. [Allowable Medications and Therapies 34](#_bookmark69)
    3. [Contraindications 34](#_bookmark70)

1. [PROTOCOL ASSESSMENTS AND PROCEDURES 35](#_bookmark71)


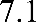
[Informed Consent 35](#_bookmark72)


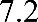
[Assessment of Eligibility 35](#_bookmark73)


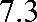
[Demographic Information 35](#_bookmark74)


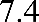
[Medical History 35](#_bookmark75)


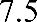
[Vital Signs 36](#_bookmark76)


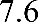
[Physical Examination 36](#_bookmark77)


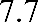
[Electrocardiogram (ECG) 37](#_bookmark78)


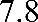
[Sequential Organ Failure Assessment (SOFA) 37](#_bookmark79)


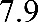
[Clinical Laboratory Assessments 38](#_bookmark81)

- - 1. [Blood Chemistry 38](#_bookmark82)
    2. [Blood Pressure 38](#_bookmark83)
    3. [Pregnancy Screen 38](#_bookmark84)


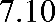
[Concomitant Medication 38](#_bookmark85)

1. [ADVERSE EVENTS 40](#_bookmark86)


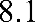
[Definition of Adverse Events 40](#_bookmark87)


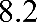
[Adverse Event Reporting 40](#_bookmark88)


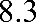
[Serious Adverse Event (SAE) 41](#_bookmark89)


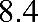
[Adverse Drug Reaction (ADR) and Suspected Adverse Reaction (SAR) 42](#_bookmark90)

[An adverse drug reaction (ADR) means any AE caused by VBI-S. 42](#_bookmark91)


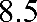
[Unexpected Adverse Reaction (UAR) 42](#_bookmark92)


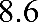
[Expected/ Anticipated Events 43](#_bookmark93)

1. [Relationship to Study Drug 44](#_bookmark94)


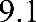
[Recording Adverse Events 45](#_bookmark95)


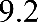
[Treatment Given as a Result of the Event 45](#_bookmark96)


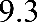
[Outcome Assessment 45](#_bookmark97)


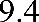
[SAE Follow-up 46](#_bookmark98)

1. [STATISTICAL ANAYLSIS 47](#_bookmark99)


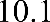
[Treatment Groups 47](#_bookmark100)


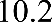
[Sample Size Consideration 47](#_bookmark101)


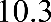
[General Statistical Considerations 47](#_bookmark102)

- - 1. [Analysis Population 47](#_bookmark103)
    2. [Missing Data 47](#_bookmark104)


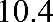
[Analysis Methods 47](#_bookmark105)

- - 1. [Interim Analysis 47](#_bookmark106)


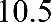
[Safety Evaluation 48](#_bookmark107)

1. [DIRECT ACCESS TO SOURCE DATA/ DOCUMENTS 49](#_bookmark108)


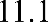
[Study Monitoring 49](#_bookmark109)


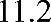
[Audits and Inspections 49](#_bookmark110)

1. [INSTITUTIONAL REVIEW BOARD 50](#_bookmark111)


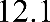
[Investigator’s Responsibilities 50](#_bookmark112)


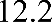
[Patient Informed Consent Requirements 50](#_bookmark113)

1. [QUALITY CONTROL AND QUALITY ASSURANCE 51](#_bookmark114)


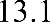
[Monitoring requirements 51](#_bookmark115)


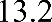
[Acceptability of electronic Case Report Forms (eCRFs) 51](#_bookmark116)


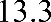
[Modification of Protocol 51](#_bookmark117)


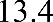
[Reporting Protocol Deviations 52](#_bookmark118)


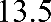
[Major Protocol Deviation and Violation 52](#_bookmark119)


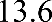
[Minor Protocol Deviation and Violation 52](#_bookmark120)

1. [ETHICS 54](#_bookmark121)


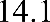
[Ethics Review 54](#_bookmark122)


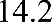
[Ethical Conduct of the Study 54](#_bookmark123)


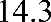
[Written Informed Consent 54](#_bookmark124)

1. [DATA HANDLING AND RECORDKEEPING 55](#_bookmark125)


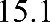
[Recording and Collection of Data 55](#_bookmark126)


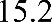
[Clinical Data Management 55](#_bookmark127)


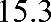
[Inspection of Records 55](#_bookmark128)


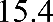
[Retention of Records 56](#_bookmark129)

1. [PUBLICATION POLICY 57](#_bookmark130)
2. [LIST OF REFERENCES 58](#_bookmark131)

# LIST OF TABLES

[Table 1: Emergency Contact Information 4](#_bookmark4)

[Table 2: Abbreviations and Specialist Terms 15](#_bookmark9)

[Table 3 Dose Regime for VBI-S 23](#_bookmark37)

[Table 4: Schedule of Assessments 24](#_bookmark38)

[Table 5: Investigational Product 30](#_bookmark51)

[Table 6: Sequential Organ Failure Assessment score 37](#_bookmark80)

# LIST OF FIGURES

[Figure 1: Mean Arterial Blood Pressure of a 39-year old woman given VBI-S after heart](#_bookmark21) [transplant 20](#_bookmark21)

[Figure 2: Results of a 69-year old woman given VBI-S after severe drop in blood](#_bookmark22)

[pressure 21](#_bookmark22)

# LIST OF ABBREVIATIONS AND DEFINITIONS OF TERMS

The following abbreviations and specialist terms are used in this study protocol.

**Table 2: Abbreviations and Specialist Terms**

| **Abbreviation or Specialist Term** | **Explanation** |
| --- | --- |
| ADL | Activities of Daily Living |
| ADR | Adverse Drug Reaction |
| AE | Adverse event |
| BUN | Blood Urine Nitrogen |
| CFR | Code of Federal Regulations |
| CRF | Case Report Form |
| CRO | Contract Research Organization |
| CS | Clinically Significant |
| CVP | Central Venous Pressure |
| DSMB | Data Safety Monitoring Board |
| ECG | Electrocardiogram |
| eCRF | Electronic Case Report Form |
| EOT | End of Treatment |
| GCP | Good Clinical Practice |
| HEENT | Head, Ears, Eyes, Nose, Throat |
| HIPAA | Health Insurance Portability Accountability Act |
| HIV | Human Immunodeficiency Virus |
| ICF | Informed Consent Form |
| ICH | International Conference on Harmonization |
| ICU | Intensive Care Unit |
| IEC | Independent Ethics Committee |
| IEC | Institutional Ethics Committee |

| IP | Investigational Product |
| --- | --- |
| IRB | Institutional Review Board |
| ITT | Intent to Treat |
| LAR | Legally Authorized Representative |
| MELD | Model for End-Stage Liver Disease |
| NCI-CTCAE | National Cancer Institute’s Common Terminology Criteria for Adverse Events |
| OAE | Other significant adverse event |
| PI | Principal Investigator |
| PT | Prothrombin Time |
| PT | Preferred Term |
| PTT | Partial Thromboplastin Time |
| SAE | Serious adverse event |
| SAF | Safety Analysis Set |
| SAP | Statistical Analysis Plan |
| SAR | Suspected Adverse Reaction |
| SOC | System Organ Class |
| SOFA | Sequential Organ Failure Assessment |
| SOP | Standard Operating Procedure |
| TEAEs | Treatment-Emergent Adverse Events |
| TPN | Total Parenteral Nutritional |
| UAR | Unexpected Adverse Reaction |
| ECHO | Echocardiography |
| LIDCO | Lithium Dilution Cardiac utput |
| NICO | Non-invasive Cardiac Output |

# INTRODUCTION

##
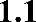
Statement of Intent

The design conduct and reporting of this study shall be conducted in compliance with the protocol, International Conference on Harmonization/Good Clinical Practice (ICH/GCP), and all appropriate regulatory requirements. The investigators participating in this study will have documented training in GCP. Independent monitoring of the trial will be accomplished utilizing a Contract Research Organization (CRO).

##
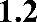
Background

Sepsis is a serious condition resulting from the presence of harmful microorganisms in the blood or other tissues and the body’s response to their presence, potentially leading to the malfunctioning of various organs, shock, and death. Inflammatory responses are activated throughout the body, which leads to microvascular blood clots and leaky blood vessels. This results in a combination of absolute hypovolemia with inadequate fluid volume in the blood vessels and/or relative hypovolemia with maldistribution of blood flow in the microcirculation due to impaired vascular responsiveness to catecholamines. This impaired blood flow, which deprives organs of nutrients and oxygen, leads to organ damage. When hypotension [systolic blood pressure < 90 mm Hg (or a drop of > 40 mm Hg from baseline) or mean arterial pressure

< 65 mm Hg] due to sepsis cannot be reversed with the infusion of fluids the patient is in a state of septic shock. ([National Institute of General Medical Sciences, 2018](#_bookmark143)).

Septic shock is associated with high morbidity and mortality with one in three patients in the hospital that die due to sepsis ([Centers for Disease Control and Prevention, 2019](#_bookmark134)). The mortality rate of septic shock ranges from 30% to 60% and affects more than a million Americans every year with an incidence rate of approximately 31 cases per 100,000 population ([Brand et al. 2017](#_bookmark133)). The condition can occur in anyone, but infants, children, elderly, men, and people with serious medical problems such as, cancer, human immunodeficiency virus (HIV), or liver disease are at higher risk of developing sepsis ([Mayr et al., 2013](#_bookmark140)).

Septic shock leads to hypovolemia due to generation of endotoxin-antibody-complement complexing macrophage activation and leukocyte lysis that results in the production of histamine, serotonin, reactive oxygen species, lysosomal enzymes, kinins and other toxic mediators. These substances induce a marked capillary permeability and a third space loss and/or highly variable blood flow in the microcirculation ([Schumer W,1984](#_bookmark142); Girardin, 1993). Fluid loss can also result from blood hemorrhage, severe burns, vomiting and diarrhea. During hypovolemia there is either an absolute volume deficit, intravascular volume is decreased, or if the blood volume is normal, there is an inadequate circulating blood volume producing decreased peripheral vascular perfusion and cellular metabolic derangements. The net result is decreased intravascular volume leading to decreased venous return to the heart, decreased stroke volume and tissue perfusion. The non-vital tissue such as gastrointestinal tract, muscle, skin and connective tissue is affected first. Later vital tissue such as brain, heart, liver and kidneys are affected.

Current standard therapy includes early detection, administration of antibiotics, removal, if possible, of the source of sepsis, and fluid resuscitation. But many cases progress beyond this early stage even with timely intervention. When the infusion of intravenous fluids is ineffective

in raising blood pressure to at least a mean of 60-65 mmHg, pressor medications such as Levophed, vasopressin, epinephrine or Giapreza (angiotensin II) must be used ([Centers for](#_bookmark134) [Disease Control and Prevention, 2019](#_bookmark134)). When on two or more pressor medications at full dose, patient’s mortality rate is 80.6% and increases to 81.9% when used at peak doses. The treatment of septic shock when pressor medications are no longer effective has been disappointing and use of extracorporeal membrane oxygenation, plasmapheresis and molecular targeted therapies have failed ([Agarwal et al., 2005](#_bookmark132)). Treatment also involves administering aggressive volume resuscitation before irreversible end-organ dysfunction occurs. The infusion of Ringer’s lactate, blood, albumin and hetastarches each has been noted to cause organ injury by a mechanism specific to each fluid.

Septic shock is defined by the ineffectiveness of fluid infusion in elevating blood pressure to survivable levels. When this occurs vasopressors must be used. In septic shock there is a hyporesponsiveness to vasopressors and in more advanced shock, a lack of effectiveness of vasopressors. There is an unmet need for a means of elevating blood pressure under the dire circumstances of advanced septic shock in which current fluids are ineffective and blood pressure cannot be elevated except with high doses of vasopressors or in which vasopressors are ineffective and there is no possibility of survival. VBI-S is being proposed to address that need.

##
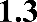
Investigational Product: VBI-S

## Description

The investigational product (IP), VBI-S, is a soybean oil emulsion comprised of as weight/volume 20% soybean oil, 2.25% glycerin and 1.2% egg lecithin with NaCl at 77 meq added on the day of use.

Egg yolk derived phospholipids are the emulsifier that envelopes the soybean fat globules and keeps them soluble in the aqueous phase. Lipid micelles are colloids with physical characteristics that render them as effective perfusion fluids as they do not readily leak out of the vascular space, and they are also able to transport oxygen and other gases.

## Proposed Mode of Action and Treatment Rationale for this study

An investigator-initiated study showed that lipid emulsions were successfully used to elevate the blood pressure of two patients who had septic shock and in whom all measures consistent with currently standard practice had failed to affect an increase in blood pressure. Failed standard of care measures included administration of normal saline, 25% albumin, vasopressin, epinephrine and Levophed (epinephrine bitartrate)

A 100% lethal mouse model for blood loss was used to compare ability of soybean oil micelles to that of Ringer’s lactate, blood and other fluids with respect to raising blood pressure and maintaining it for one hour ([Armbruster et al., 2013](#_bookmark132)). Soybean oil emulsions were superior to other fluids in restoring and maintaining blood pressure. The oxygen carrying ability of soybean oil micelles exceeded that of water and off-loading of oxygen from the micelles was nearly as fast as that from off-loading from water. Nitric oxide also preferentially loaded onto soybean oil micelles. Animals that received soybean oil emulsions did not exhibit fat embolization.

The proposed clinical trial is a first in man study to evaluate the safety and efficacy of VBI-S in

elevating the blood pressure of septic shock patients with absolute or relative hypovolemia. This study provides opportunity for patients to have supervised treatment with VBI-S. Patients participating in this study will contribute to the development of a new drug which has the potential to become a treatment option for them and others in the future. Currently, after fluids and vasopressors have failed to elevate blood pressure, there is no alternative therapy.

## Rationale for Dose Selection

In the investigator-initiated study, two patients were given the prototype of VBI-S 500ml infused over 30 minutes. The proposed dosing regimen is designed to allow for patient variability of blood volume and vascular unresponsiveness to catecholamines in which some may require less and others more to achieve the goal to reach the mean blood pressure of 60-65 nmHg and/or an elevation of at least 10 mmHg in the mean blood pressure.

## Previous Clinical Experience

This is the first human study for the development of VBI-S in elevating the blood pressure in septic shock patients with absolute or relative hypovolemia.

The 20% soybean oil emulsion used for VBI-S has a long history of clinical use in patients as Intralipid 20% for total parenteral nutritional (TPN) support in both adult and pediatric populations. Intralipid^®^ 20% has been approved by the FDA for TPN since 1972 ([Hansen et al.,](#_bookmark137) [1976](#_bookmark137)) in both adult and pediatric populations, including neonates ([Kabi F, 2017](#_bookmark139)) ([Kabi F, 2015](#_bookmark138)). Intravenous infusion of lipids has also been used off-label as an antidote for poisoning or drug overdose caused by lipophilic agents ([Ciechanowicz et al., 2011](#_bookmark135)) ([Weinberg, 2012](#_bookmark145)). The FDA has approved the infusion of volumes of lipid as Intralipid^®^ 20% similar to those proposed in this clinical trial for parenteral nutrition. There have been two instances of patients with septic shock in whom currently available fluids and medications given to elevate their blood pressures to a survivable level had failed. The patients were then treated with a 500 ml prototype of VBI-S followed by successful elevation of blood pressure. There were no adverse effects.

**Case #1:** A 39-year-old woman who had severe heart failure and was given a heart transplant. Unfortunately, within hours after transplantation surgery her new heart failed. Cardiac and pulmonary assist devices were inserted in order to give the new heart and lungs time to recover. She developed septic shock as evidenced by the cultures of her blood that grew out gram positive bacteria. This was treated with intravenous fluids and broad-spectrum antibiotics. As she appeared to improve the assist devices were removed. In the initial hours she did well. But soon her blood pressure markedly decreased, and she was given four liters of fluid that failed to elevate her blood pressure. She later went into cardiac arrest. Spontaneous cardiac contraction was restored after open cardiac massage in which epinephrine was injected directly into her heart. After this event her blood pressure was only 50/40 with a mean of 43 mmHg despite being on high dose Levophed. Since all measures had failed to elevate her blood pressure 500 ml of Intralipid 20% was infused. This resulted in an increase in blood pressure as shown in [Figure 1](#_bookmark21). The blood pressure remained elevated for approximately 145 minutes. In addition, during this time the oxygen saturation improved from 90-91% before the infusion to 97-98% after the infusion without any change in the ventilator settings. When the family decided to withdraw care the blood pressure decreased.

## Figure 1: Mean Arterial Blood Pressure of a 39-year old woman given VBI-S after heart transplant


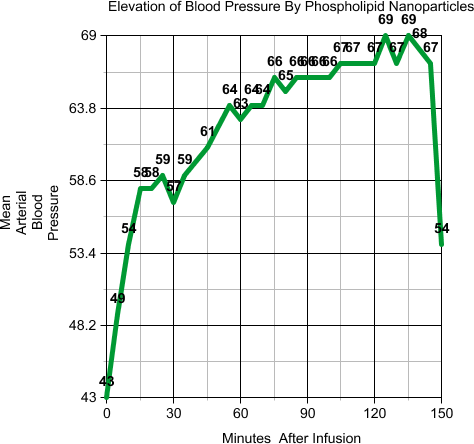


**Case #2** This patient, WB was a 69-year-old woman. Her source of infection was a gangrenous and purulent foot. She had comorbidities that included diabetes mellitus, end stage renal disease requiring hemodialysis, and peripheral vascular disease. Normal saline and 25% albumin had failed to elevate her blood pressure. She was on a combination of vasopressors at the extraordinarily high doses of Levophed at 100μg/minute, vasopressin at 0.1 unit/minute and epinephrine at 30 μg/minute. In spite of these measures her blood pressure was only 61/28. In order to elevate her blood pressure to survivable levels the pharmacy was asked to prepare a solution of Intralipid 10% and NaCl at a concentration of 77 mM. 500 ml of this mixture was given to her intravenously. After this infusion her blood pressure increased from 61/28 (mean = 39) to 91/43 (mean = 59; [Figure 2](#_bookmark22)). Unlike the previous case WB’s family did not decide to withdraw care until over 14 hours after the PN infusion. [Figure 2](#_bookmark22) is a graph of blood pressure over time.

From the graph one can see the elevation of blood pressure after the prototype was given. After this initial increase the patient was started on dialysis and the pressure decreased. Dialysis removed some of the prototype and blood from her circulation. Dialysis was stopped and blood with the prototype was returned to her intravascular space. Her blood pressure rose again and stayed up until around 14:00 on the next day when her family decided to withdraw care. In addition, after the infusion of the prototype her oxygenation improved, and we were able to decrease the concentration of oxygen given to her via the ventilator from 70% to 50%. One measure of oxygenation is the ratio of the partial pressure of oxygen in the arterial blood to the percentage of oxygen given via the ventilator. Before receiving the prototype, this ratio was only

1.31. After the prototype had been given on the next day the ratio increased to 2.35. In both cases the oxygen saturation increased after the infusion. There were no adverse effects of giving the prototype.

**Figure 2: Results of a 69-year old woman given VBI-S after severe drop in blood pressure**


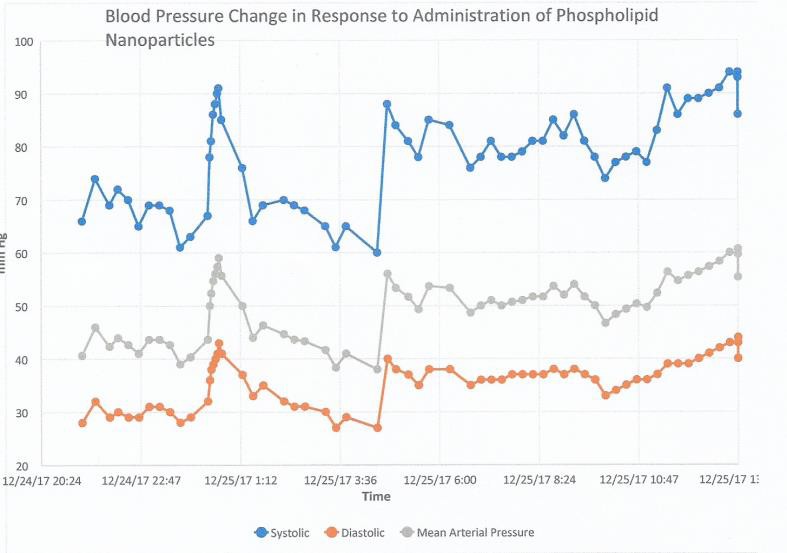


## Risks and Benefits Assessment

## Potential Benefits

Soybean oil, the major lipid component in VBI-S contains the antioxidant vitamin E ([Clemente](#_bookmark136) [et al., 2009](#_bookmark136)) ([Mejean et al., 2015](#_bookmark141)) that may provide some clinical benefit ([Singh et al., 2007](#_bookmark144)). Patients participating in this study will contribute to the development of a new drug which has the potential to become a treatment option for them and others in the future.

## Potential Risks

VBI-S is comprised of 20% soybean oil, glycerin at 2.25% and egg lecithin at 1.2% and contains aluminum that may be toxic and may reach toxic levels with prolonged administration if kidney function is impaired. Other potential risks include immediate or early adverse reactions, each of which has been reported to occur in clinical trials, in an incidence of less than 1%; dyspnea, cyanosis, allergic reactions, hyperlipemia, hypercoagulability, nausea, vomiting, headache, flushing, increase in temperature, sweating, etc. delayed adverse reactions such as hepatomegaly, jaundice due to central lobular cholestasis, splenomegaly, thrombocytopenia, leukopenia, transient increases in liver function tests, and overloading syndrome.

# STUDY OBJECTIVES AND ENDPOINTS

##
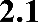
Primary Objective(s)

The primary objective of this study is to evaluate the safety and efficacy of VBI-S in elevating the blood pressure in septic shock patients with absolute or relative hypovolemia.

##
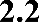
Secondary Objective(s)

The secondary objective of this study is to determine the use of pressor drugs after infusion of VBI-S.

##
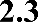
Study Endpoints

## Primary Endpoints

The primary endpoint of this study is the proportion of patients in whom mean blood pressure increased by at least 10 mmHg with a mean blood pressure target of 60-65 mmHg.

## Secondary Endpoints

The secondary endpoint of this study is the proportion of patients in whom the dose of pressor drugs could be decreased after infusion of VBI-S to maintain a mean arterial pressure of 60-65 mmHg.

## Exploratory Endpoints

The tertiary and exploratory endpoints of this study are:

- - - 1. Proportion of patients that need the change in the dose and type of vasoactive drugs post administration of VBI-S infusion
      2. 28-day mortality post-administration of VBI-S infusion
      3. Duration of ICU stay
      4. Duration of hospital stay
      5. Proportion of patients with new infections after receiving VBI-S
      6. Change in volume status as indicated by the central venous pressure (CVP), ultrasound assessment of the inferior vena cava or internal jugular vein, or changes in stroke volume index or cardiac output
      7. Change in Sequential Organ Failure Assessment Score
      8. Change in Arterial Oxygen saturation and partial pressure of oxygen and carbon dioxide, base excess post‐infusion
      9. Change in serum lactate
      10. Change in platelet count
      11. Change in Prothrombin Time (PT) and INR
      12. Change in Partial Thromboplastin Time (PTT)
      13. Change in serum sodium, potassium, chloride, HCO3, CBC, blood urine nitrogen (BUN) creatinine, liver function tests, amylase, lipase, and troponin.
      14. Change in plasma triglycerides

# STUDY DESIGN

##
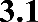
Overall Study Design

The aim of this Phase IIa, proof of concept, open label study is to evaluate the safety and efficacy of VBI-S in elevating the blood pressure of septic shock patients with absolute or hypovolemia and who have a Sequential Organ Failure Assessment (SOFA) score ≥5. Blood pressure will be measured by a catheter in the arterial system or a blood pressure cuff whenever an arterial line is not available.

##
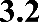
Number of Patients

20 patients will be enrolled in this trial.

##
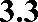
Treatment Assignment

This study is designed as an open-label study. All patients will receive an intravenous infusion administration of VBI-S. All study personnel and patients will have knowledge of the treatment assignments after enrollment and throughout the study.

After the 15-minute observation period to ensure that the blood pressure baseline is stable, VBI- S will be given in continuously increasing volumes at a rate of 999 ml/hour to a maximum of 1500 ml of VBI-S with a goal to have an increase in the mean blood pressure of at least 10 mmHg or more. Repeat boluses will be given as needed to maintain an elevation of blood pressure of 10 mmHg or more and a MAP of 60-70 for a 48-hour trial period.

The dosing regimen is shown in [Table 3](#_bookmark37). The blood pressure goal will be an increase in the mean blood pressure of at least 10 mmHg or more. Repeat boluses will be given as needed to maintain an elevation of blood pressure of 10 mmHg or more for a 48-hour trial period.

## Table 3 Dosing Regime for VBI-S

| Dose level | VBI-S dose | Rate |
| --- | --- | --- |
| 0* (Starting dose) | 100 ml | 999 ml/hour |
| 1 | 200 ml Total= 300 ml |  |
| 2 | 400 ml Total=700 ml |  |
| 3  (Maximum dose of a single bolus per 24 hours) After 24 hours more doses may be given | 800 ml Total= 1500 ml |  |

The Schedule of Assessments is described in [Table 4](#_bookmark38).

Protocol No: VBI-S-01 Version: 3.0/5 Nov 2021

## Table 4: Schedule of Assessments

| Tests and Evaluations | Screening phase | Baseline Phase | Treatment phase | Follow-up phase |
| --- | --- | --- | --- | --- |
|  | Screening | Pre-Infusion^1^ | 48 hour Trial Period-ICU | 28 days-Hospital Ward |
| Informed Consent | X |  |  |  |
| Eligibility criteria | X |  |  |  |
| Demographics; Medical History | X |  |  |  |
| Physical Exam |  | X |  | X3 |
| Vital Signs^7^ |  | X | X6 | X5 |
| SOFA score | X |  | X | X |
| Dose of Vasopressors |  | X | X6 | X |
| Pregnancy test | X |  |  |  |
| ECG (12-Lead) | X |  |  |  |
| Fraction of inspired oxygen |  | X | X6 |  |
| VBI-S Administration |  |  | X |  |
| CVP [Optional] |  | X2 | X6 |  |
| Blood Samples^10^ |  | X | X4 | X9 |
| Urine output |  | X8 | X6 | X5 |
| AEs and Concomitant Medications | X | X | X | X |

1. 15 minute pre-infusion observation period.
2. If the patient does not have one, an arterial line and central venous catheter will be placed prior to the 15-minute pre-infusion observation period. The central venous line is optional if another means of accessing intravascular volume is available. These other means such as a Swan Ganz catheter, Ultrasound, esopahageal doppler, NICO or other published methods can be used to rule out hypervolemia If placing the arterial line is not possible or would not be optimal for the patient then a blood pressure cuff may be used to measure blood pressure.
3. Every seven days a physical examination will be performed.
4. Blood sample will be drawn daily
5. At least every four hours measurements of blood pressure, pulse, temperature, respiratory rate and oxygen saturation, heart rate and urine output
6. If monitors available, blood pressure should be taken every 15 minutes after the initial elevation of the MAP by 10 mmHg using VBI-S. Dose of vasopressors every 15 minutes should also be recorded. There should also be hourly measurements of blood pressure, pulse, temperature, dose of vasopressors, CVP (if available) respiratory rate, oxygen saturation, fraction of inspired oxygen and urine output
7. Vital signs include blood pressure, respiratory rate and oxygen saturation, pulse, heart rate, and body temperature
8. Urine output over previous two hours
9. Blood sample at least every seven days for twenty-eight days after the initial infusion of VBI-S.
10. Blood sample for arterial blood gas sodium, potassium, chloride, HCO3, BUN, creatinine, alanine aminotransferase, aspartate aminotransferase, alkaline phosphatase, total, direct, and indirect bilirubin, amylase, lipase, troponin, prothrombin time, international normalized ratio, partial thromboplastin time, triglycerides, lactic acid and procalcitonin.

##
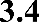
Dose Adjustment Criteria

## Safety Criteria for Adjustment or Stopping Doses

Patients will be routinely monitored for adverse events by monitoring vital signs, physical/neurological examinations, clinical chemistry, hematology, urinalysis, and ECGs.

##
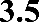
Criteria for Study Termination

## Early discontinuation of this study

The investigators may terminate this study for a reasonable cause, provided a written notice is submitted within a reasonable time in advance of the intended termination.

If a drug-related severe local reaction or SAE occurs at any time during the study, the DSMB will review the case immediately. The study will be immediately suspended, and no additional VBI- S doses will be administered pending review and discussion of all appropriate study data by the DSMB. The study investigators will make an assessment that the SAE is possibly, probably, or definitely related to VBI-S based upon close temporal relationship or other factors.

The study will not be restarted until all parties have agreed to the course of action to be taken and the Institutional Review Board/International Ethic Committee (IRB/IEC) (s) has/have been notified.

## Discontinuation of individual patient

Patients will be withdrawn from the study for any of the following reasons:

- - - - Withdrawal of informed consent
      - Disease progression (at the discretion of the PI)
      - Unacceptable toxicity
      - Changes in the patient's condition which render the patient unacceptable for further treatment in the judgment of the PI
      - Patient is lost to follow-up

Patients will also to be withdrawn at any time if the investigators concludes that it would be in the patient's best interest for any reason. Protocol violations do not lead to patient withdrawal unless they constitute a significant risk to the patient’s safety.

Patients can withdraw by themselves or by their legally authorized representative (LAR) from the trial for any reason at any time. They are to be considered withdrawn if they state an intention to withdraw for any reason, or if any of the following occurs:

- - - - Discovery of patient ineligibility
      - Missed / unscheduled / incomplete / incorrect assessments that result in patients being put at risk.

The investigators must determine the primary reason for a patient’s withdrawal from the study and record this information on the eCRF.

##
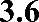
Data Safety Monitoring Board (DSMB)

A Data Safety Monitoring Board (DSMB) will review the study data. The DSMB will monitor the safety of the trial from the beginning. The DSMB will consist of at least three members (combination of physicians/critical care nurses/regulatory experts) who will review all individual physiological variables and the safety laboratory values, all adverse events (AE) including serious AEs as well as unrelated and all related deaths during the Treatment and Follow-Up Phases. All utilized vasopressors used and their doses in each patient and the patient’s vitals at the time will also be provided. All expedited safety reports will be provided in real time to the DSMB chair upon being reported to FDA.

A further description of the DSMB reporting requirements, meeting frequency etc. can be found in the DSMB charter.

# SELECTION AND WITHDRAWAL OF PATIENTS

##
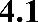
Patient Inclusion Criteria

- 1. Male or female at least 18 years of age
  2. Evidence of bacterial infection demonstrated by positive blood culture and/or a known source of infection or an elevated procalcitonin ≥ 2 ng/ml
  3. Patient has a mean blood pressure < 65 mmHg that is unresponsive to fluids currently available on the market.
  4. Sequential Organ Failure Assessment (SOFA) score ≥ 5.
  5. Sepsis diagnosis: The presence of infection which can be proven or suspected by 2 or more of the following criteria:
     - Lactate > 2 mmol/L
     - Mottled skin
     - Decreased capillary refill of nail beds or skin
     - Fever > 38.3˚C, or 101˚F
     - Hypothermia < 36 ˚C core temperature (<96.8˚F)
     - Heart rate > 90
     - Tachypnea
     - Change in mental status
     - Significant edema or positive fluid balance (>20 mL/kg over 24 hours)
     - Hyperglycemia (>140 mg/dL) in someone without diabetes
     - White blood cell count > 12,000 or less than 4,000, or with >10% "bands" (immature forms)
     - Elevated C-reactive protein in serum (according to your lab's cutoffs)
     - Elevated procalcitonin in serum (≥ 2 ng/ml)
     - Arterial hypoxemia (PaO2/FiO2 < 300)
     - Acute drop in urine output (<0.5 ml/kg/hr for at least 2 hours despite fluid resuscitation, or about 30 ml/hour for a 70 kg person)
     - Creatinine increase > 0.5 mg/dL
     - INR > 1.5 or aPTT > 60 seconds
     - Absent bowel sounds (ileus)
     - High bilirubin (total bilirubin > 4 mg/dL

##
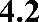
Patient Exclusion Criteria

1. Patients with a ventricular assist device
2. Acute coronary syndrome
3. Pregnant
4. Bronchospasm
5. Mesenteric ischemia
6. Emergency surgery
7. Acute liver disease (Hepatitis B and C as examples)
8. Liver failure with a Model for End-Stage Liver Disease (MELD) score ≥ 19
9. Hematologic or coagulation disorders including thrombocytopenia (platelet count

<50,000) and associated with hemodynamically significant active bleeding.

1. Absolute neutrophil count of < 1000 mm3
2. Current participation or participation in another experimental or device study within the last 30 days before the start of this study. Patient may be included if on other drugs for COVID-19 and or septic shock
3. Patients with a known allergy to soybeans or eggs
4. Patient is hypervolemic as assessed by CVP, ultrasound, Swan Ganz catheter, Flo-Trac, esophageal doppler, bioimpedance, ECHO, Partial carbon dioxide rebreathing (NICO), lithium dilution (LIDCO) or other method published in a peer reviewed journal

##
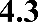
Patient Withdrawal Criteria

A patient who enters the Treatment Phase but does not complete the study is considered to have prematurely withdrawn from the study.

At any point during the study all patients have the right to withdraw without prejudice to future care. Documentation to whether each patient completed the clinical study will be recorded. If for any patient, study treatment was discontinued, the reason(s) will be documented.

The investigators can discontinue a patient at any time if it is considered medically necessary.

In addition, patients WILL be withdrawn from the study, in consultation with the Medical Monitor and the investigators, if any of the following are met:

- - Patient is significantly non-compliant with the requirements of the protocol.
  - Investigators determine that it is in the best interest of the patient.
  - Patient chooses to withdraw consent
  - Patient experiences an adverse event
  - Patient becomes pregnant
  - Lost to follow-up
  - Death
  - Discontinuation of study by Sponsor

Premature withdrawal from the study MAY occur if, in consultation with the Medical Monitor and the investigators, any of the following are met:

- - A patient is treated with a prohibited medication.
  - Major protocol violation

The investigators or study team must notify the sponsor when a patient has been discontinued due to any adverse event or complication. When a patient discontinues/withdraws prior to study completion, all applicable activities scheduled for the End of Study (EOS) visit should be performed at the time of discontinuation.

## Data Collection from Withdrawn Patient

Patients or a legally authorized representative (LAR) may withdraw from the study or discontinue study treatment at any time; however, the sponsor is dedicated to minimizing missing data in this study. An excessive rate of withdrawals can render the study non-evaluable; therefore, unnecessary withdrawal of patients should be avoided. However, patients must be removed if a safety concern occurs that is regarded as clinically relevant by the investigators. Should a patient decide to withdraw, every effort will be made to complete and report the observations as thoroughly as possible.

All assessments scheduled for the End of Treatment (EOT) visit must be performed if a patient discontinues treatment before the EOT visit. A patient prematurely ending active study treatment will enter the regular follow-up period.

If a patient discontinues during the follow-up period, he/she should come in for one final follow-up visit and every attempt should be made to collect survival data over the phone.

The investigators can discontinue a patient at any time if in his clinical judgment considers to be medically necessary. Investigators considering discontinuing study treatment should contact the medical monitor prior to such discontinuation. Patients who have study treatment discontinued will continue to be followed, per protocol, whenever possible. Patients who have study treatment discontinued due to a serious adverse event (SAE) will be followed until resolution or stabilization of the event.

If a patient is withdrawn from the study at any time due to an adverse event or SAE, the procedures stated in [Section](#_bookmark88) [8.2](#_bookmark88), respectively must be followed.

Every attempt should be made to collect follow-up information. The reason for withdrawal from the study will be recorded in the source documents and on the appropriate page of the eCRF. Before a patient is identified as lost-to-follow up, the site should make all reasonable efforts to contact the patient. These attempts must be documented and should include at a minimum one phone call and one certified letter.

# STUDY TREATMENT

Procedures to be performed during the study period are described below and provided as Schedule of Assessment in [Table 4](#_bookmark38).

A description of the Study Drug is provided below in [Table 5.](#_bookmark51)

## Table 5: Investigational Product

|  | **Investigational Product** |
| --- | --- |
| **Product Name:** | VBI-S |
| **Dosage Form:** | Fat emulsion |
| **Formulation** | 20% Soybean oil emulsion |
| **Unit Dose** | Volume needed to achieve an increase in MAP of 10 mmHg up to 1500 ml |
| **Route of Administration** | Intravenous Infusion |
| **Physical Description** | White liquid |
| **Manufacturer** | Latitude Pharmaceuticals  under contract with Vivacelle Bio, Inc. |

##
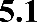
Treatment Compliance

The investigators will dispense the study medication only for use by patients enrolled in the study as described in this protocol. The study medication is not to be used for reasons other than those described in this protocol.

##
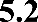
Study Schedule

## Screening Phase

Screening phase is designed to determine whether patients are eligible to proceed to the Treatment Phase of the study. This visit consists of a series of assessments designed to determine eligibility. A written informed consent from the patient or LAR will be obtained by the investigators or suitably qualified individual before the performance of any protocol- specific procedure.

The patient or LAR will sign and date the informed consent form (ICF) and Health Insurance Portability Accountability Act (HIPAA) authorization (according to site policy and practices) prior to any study-related procedures. A unique identification number (screening number) will be assigned to each patient who has provided written informed consent. The patient screening number will incorporate a two-digit region code (01, 02 or 03...), a three-digit Study Centre number (001, 002 or 003….) and a three-digit numeric ID assigned in successive order of

entering the study after signing the ICF at each center, beginning with 001 at each site (e.g. 01- 001-001).

| **Patient Screening #:** | **XX** | **-** | **YYY** | **-** | **ZZZ** |
| --- | --- | --- | --- | --- | --- |
| XX=Region | | YYY=Study Centre | |  | ZZZ=Patient Numeric ID |

All study centers will be instructed to maintain the study-specific pre-screening, screening and enrolment logs at their sites

Prior to all screening procedures, the investigators are responsible for explaining all aspects of this trial to the LAR including potential side effects and alternative treatments available. The investigators must answer all questions by the LAR according to his/her best knowledge and must give the LAR ample time to consider participation. If LAR grants participation in this trial, he/she must first give written informed consent. Only then may the investigators proceed with the screening procedure.

The following evaluations and examinations have to be done prior to administration of study treatment, if not stated otherwise:

- - - - Signed informed consent
      - Eligibility criteria
      - Demographic information
      - Disease history, including date of first diagnosis and the history of the course of the disease
      - Medical and surgical history, including past or concurrent clinically significant disease(s)
      - Previous and concomitant medication
      - Adverse events
      - Physical examination
      - SOFA score
      - 12- Lead Electrocardiogram (ECG)
      - Pregnancy test, if female of childbearing potential (serum or urine)

All screening information will be fully documented in the patient’s medical records (i.e., source documents).

- For consented patients who do not meet eligibility criteria, a Screen Failure electronic Case Report Form (eCRF) will be completed. The Screen Failure eCRF will contain the following details: the patient identification number, the date of ICF signature, demographic information, and the reason for screen failure. No additional information will be required for patients who fail screening.
- For consented patients who meet eligibility criteria, all required screening information will be transcribed onto the appropriate page of the eCRF.

## Baseline Phase: 15- minute pre-infusion observation period

The patient will have an arterial line and central venous catheter placed prior to the 15-minute pre-infusion observation period. In some cases, the placement of an arterial line will unduly delay the treatment of the patients’ hypotension when a survivable blood pressure cannot be achieved with conventional fluids and vasopressors. In such cases blood pressure can be monitored with an automatic or manual sphygmomanometer.

Within 15 minutes prior to the infusion of VBI-S a baseline physical examination will be conducted as described in [section 7.6.](#_bookmark77) In addition, study parameters such as blood pressure, pulse, temperature, dose of vasopressors, CVP, respiratory rate and oxygen saturation, fraction of inspired oxygen, AEs and concomitant medications will be recorded. The urine output for the two previous hours will be recorded. Blood will be drawn for arterial blood gas sodium, potassium, chloride, HCO3, BUN, creatinine, glucose, alanine aminotransferase, aspartate aminotransferase, alkaline phosphatase, total, direct, and indirect bilirubin, amylase, lipase, troponin, prothrombin time, international normalized ratio, partial thromboplastin time, triglycerides, lactic acid and procalcitonin.

## Treatment Phase: Intensive Care Unit (ICU)-48- hour trial period.

After the 15-minute observation period, VBI-S will be given in continuously increasing volumes at a rate of 999 ml/hour to a maximum of 1500 ml of VBI-S. Repeat boluses will be given as needed to maintain an elevation of blood pressure of 10 mmHg or more or as needed to maintain a mean blood pressure of 60-70 mmHg for a 48-hour trial period.

While the patient is in the intensive care unit hourly measurements of blood pressure, heart rate pulse, temperature, dose of vasopressors, CVP, respiratory rate and oxygen saturation, fraction of inspired oxygen and urine output, AEs and concomitant medications will be recorded.

While the patient is in the intensive care unit blood will be drawn daily for arterial blood gas sodium, potassium, chloride, HCO3, BUN, creatinine, alanine aminotransferase, aspartate aminotransferase, alkaline phosphatase, total, direct, and indirect bilirubin, amylase, lipase, troponin, prothrombin time, international normalized ratio, partial thromboplastin time, triglycerides, lactic acid and procalcitonin.

## Follow-up Phase: Hospital Ward-28 days

While on the hospital ward at least every four hours measurements of blood pressure, pulse, temperature, respiratory rate and oxygen saturation, heart rate, and urine output, AEs and concomitant medications will be recorded.

In addition, measurements of arterial blood gas, sodium, potassium, chloride, HCO3, BUN, creatinine, alanine aminotransferase, aspartate aminotransferase, alkaline phosphatase, total direct and indirect bilirubin, amylase, lipase, troponin, prothrombin time, international normalized ratio, partial thromboplastin time, triglycerides, lactic acid and procalcitonin at least every seven days for twenty-eight days after the initial infusion of VBI-S.

Every seven days a physical examination will be conducted as described in [section 7.6](#_bookmark77)

# STUDY DRUG MATERIALS AND MANAGEMENT

##
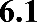
Study Drug

## Description of Investigational Product

The investigational product (IP), VBI-S, is a soybean oil emulsion comprised of n a 20% soybean oil lipid emulsion with NaCl at 77 meq added on the day of use for elevating blood pressure in septic shock patients with absolute or relative hypovolemia.

## Drug Substance

The drug substance is a soybean oil emulsion comprised of n a 20% soybean oil lipid emulsion with NaCl at 77 meq added on the day of use.

## Drug Product

The drug product is identical to the drug substance.

## Drug Packaging and Labeling

VBI-S will be supplied to the clinical sites in sterile bottles. Study treatment will be labeled, according to the regulatory guidelines, as an investigational product to ensure that the IP will not be used outside of the clinical investigation. The Sponsor name, protocol number, and any additional relevant information will appear on the pack label.

**Investigational Product**

**450 mL**

**VBI-S**

**Protocol No: VBI-S-01 Storage Condition: Below 25 ºC**

**Investigator:**

**Patient Number:**

**Vivacelle Bio, Inc.**

**Caution: Investigational Drug – Limited by Federal law to investigational use only**

## Drug Storage and Handling

VBI-S should be stored at below 25°C (77° F). Do not freeze VBI-S. If accidentally frozen, discard the bottle.

##
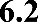
Investigational Product Administration

VBI-S will be given intravenously in continuously increasing volumes up to 1500 ml at a rate of 999 ml/hour in any single bolus.

##
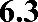
Investigational Product Accountability

The investigators or study staff will verify the integrity of the clinical trial supplies (storage conditions, correct amount received, condition of shipment, kit numbers, etc.) according to their standard operating procedures. The following data will be tracked on the drug accountability log provided by the Sponsor, and recorded in the eCRF:

- - - - Date received
      - Lot number
      - Date dispensed
      - Patient number

##
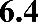
Concomitant Medications and Contraindication

## Prohibited Medicine and Therapies

The following medications and therapies are prohibited during the study:

- - - - Any other investigat ional drug

## Allowable Medications and Therapies

Allowable medications and therapies will be those medications/therapies that are not otherwise prohibited and, in the judgment of the investigators, are required for proper medical care.

## Contraindications

VBI-S is contraindicated in patients with disturbances of normal fat metabolism such as pathologic hyperlipemia, lipoid nephrosis or acute pancreatitis if accompanied by hyperlipidemia.

# PROTOCOL ASSESSMENTS AND PROCEDURES

##
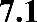
Informed Consent

A written informed consent will be obtained for this study by the investigators or designee from all patients prior to performance of any protocol-specific procedure. This study will be conducted in accordance with the provisions of the Declaration of Helsinki.

The investigators must comply with applicable regulatory requirements and must adhere to the Good Clinical Practice (GCP) in the process of obtaining and documenting the informed consent. The investigators, or designee, must also inform patients of all pertinent aspects of the study. The written consent will be obtained from the patient. In case the patient is unable to give consent, it will be obtained from the LAR.

Before written informed consent is obtained from the LAR, the investigators or a person designated by the investigators, must provide the LAR enough time and opportunity to inquire about the details of the study and to decide whether or not to participate in the trial. All questions addressed by the LAR about the study must be answered to the satisfaction of the LAR. Prior to the patient’s participation in the trial, the written informed consent must be signed and personally dated by the LAR. Authorization for release of protected health information must also be obtained, as per local policies.

##
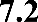
Assessment of Eligibility

The investigators must assess patient’s continued eligibility for the study as per the Inclusion and Exclusion criteria, during the Screening Phase. The eligibility criteria are described in [Section 4.1](#_bookmark46) (Inclusion Criteria) and [Section 4.2](#_bookmark47) (Exclusion Criteria).

In the event that the patient is not suitable or eligible for the study, the patient will be considered “screen failure”.

##
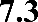
Demographic Information

For the purposes of this study, demographic information will include:

- Dates of ICF signature
- Date of birth
- Gender
- Race (American Indian/Alaskan Native, Asian, Black/African American, Caucasian, Native Hawaiian/Pacific Islander, or other)
- Ethnicity (Hispanic/Latino or Not Hispanic/Latino)

##
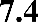
Medical History

Medical history will be assessed at screening and will include the following:

- All ongoing medical conditions
- All previously resolved medical conditions that are relevant in the judgment of the Investigator
- Any prior medical conditions that have resolved within the last year

Events that emerge prior to the administration of VBI-S will be recorded in the medical history and not as AEs. Aside from being used to determine patient eligibility, this information will permit the investigators to record the nature, duration and severity of any ongoing baseline medical conditions prior to the patient’s receiving IP treatment.

Medical history will be recorded using the body system categories outlined below:

Cardiovascular Lymphatic

Respiratory Hematologic

Gastrointestinal Immunologic

Renal Dermatologic

Hepatic Psychiatric

Neurological Genitourinary+

Endocrine Other

For each relevant history, the following will be documented:

- Disease/disorder/condition
- Date of diagnosis
- History status (resolved or ongoing)

##
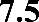
Vital Signs

All vital sign findings will be listed and summarized.

- Vital signs include:
  - Blood pressure (supine),
  - Heart rate
  - Respiratory Rate and oxygen saturation
  - Pulse
  - Body temperature (oral, axillary, tympanic or rectal)

##
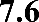
Physical Examination

A complete physical examination will be performed to include a review of all systems. Each body system will be classified as being either normal or abnormal, with abnormalities for each body system noted. Subsequent physical examinations will identify changes from the baseline examination with both positive and negative changes being noted. Each abnormality will be recorded, and the investigators will record an assessment of its clinical significance.

The complete physical examination will include routine examinations for the following:

- General Appearance
- Head, Ears, Eyes, Nose, Throat (HEENT)
- Lymph Nodes
- Heart/Cardiovascular abnormalities
- Respiratory
- Abdomen
- Genitourinary
- Musculoskeletal and Extremities
- Neurologic abnormalities Dermatologic abnormalities
- Any other body system for which an abnormality is noted and which, in the opinion of the Investigator, is relevant to the safety of the patient or could impact safety or efficacy results for the patient; i.e., the abnormality is clinically significant (CS).

##
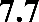
Electrocardiogram (ECG)

A 12-lead ECG will be performed at the visits as shown in the Study Plan to monitor changes in cardiac conduction. An ECG should also be performed in the event of any cardiac AE.

##
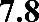
Sequential Organ Failure Assessment (SOFA)

The Sequential Organ Failure Assessment is used to predict the morality in adult sepsis and septic shock patients admitted to intensive care unit ([Table 6](#_bookmark80)).

## Table 6: Sequential Organ Failure Assessment score

| **SOFA score** | **1** | **2** | **3** | **4** |
| --- | --- | --- | --- | --- |
| Respiration | | | | |
| PaO2/FIO2 (mm Hg) | <400 | <300 | <220 | <100 |
| Coagulation | | | | |
| Platelets ×10^3^/mm^3^ | <150 | <100 | <50 | <20 |
| Liver | | | | |
| Bilirubin (mg/dL) | 1.2-  1.9 | 2.0-5.9 | 6.0-11.9 | >12.0 |
| Cardiovascular | | | | |
| Hypotension | MAP  <70 | Dopamine ≤5 or dobutamine (any) | Dopamine >5 or norepinephrine  ≤0.1 | Dopamine >15 or norepinephrine  >0.1 |
| Neurological | | | | |
| Glasgow Coma Score | 13-14 | 10-12 | 6-9 | <6 |
| Renal | | | | |
| Creatinine (mg/dL) or urine output (mL/d) | 1.2-  1.9 | 2.0-3.4 | 3.5-4.9 or <500 | >5.0 or <200 |

The following calculator will be used to determine SOFA score

https://clincalc.com/IcuMortality/SOFA.aspx

##
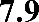
Clinical Laboratory Assessments

All procedures will be performed following the standard operating procedure (SOP) of the laboratory used.

## Blood Chemistry

A blood sample will be taken to evaluate arterial blood gas sodium, potassium, chloride, HCO3, BUN, creatinine, glucose, alanine aminotransferase, aspartate aminotransferase, alkaline phosphatase, total, direct, and indirect bilirubin, amylase, lipase, troponin, prothrombin time, international normalized ratio, partial thromboplastin time, triglycerides, lactic acid and procalcitonin.

## Blood Pressure

Blood pressure will be monitored every hour during the 48-hour trial period in ICU and then every four hours during the 28-day hospital ward stay after the treatment. Along with blood pressure at these time points, blood pressure, pulse, temperature, respiratory rate and oxygen saturation, heart rate and urine output will also be measured.

## Pregnancy Screen

A urine or serum pregnancy test for women of childbearing potential. This test will be performed using a commercially available kit.

##
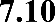
Concomitant Medication

The patient may receive any medications judged necessary by the investigators, provided such medications are not listed in [Section 6.4.1.](#_bookmark68)

All medications and therapies (including over-the-counter and prescription medicine, vitamins or/and herbal supplement) administered or taken by the patient beginning 30 days prior to Screening Phase and throughout the study will be recorded in the source documents and on the appropriate page of the electronic Case Report Form (eCRF).

For each medication and non-study treatment, the following will be documented:

- - - - Medication/treatment name (generic name may be used if trade name is unknown)
      - Dose, unit, and frequency of dosing (individual dosages, not total daily dose).

Note: Each new dose of medication should be recorded as a separate entry, with the exception of medications that are given on a sliding scale. For these, it is acceptable to enter the range of the dosage, including the start and stop dates for which the specified dosage range was used.

- - - - Route of dosing
      - Indication for use
      - The start date
      - The stop date (if medication/therapy is not ongoing)

# ADVERSE EVENTS

The investigators are responsible for the detection and documentation of events meeting the criteria and definition of an AE or SAE, as provided in this protocol. During the study when there is a safety evaluation, the investigators or site staff will be responsible for detecting, documenting, and reporting AEs and SAEs as detailed in this Section of the protocol.

##
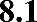
Definition of Adverse Events

An AE is defined as any untoward or unfavorable medical occurrence associated with the patient’s participation in the research, whether or not considered related to the patient’s participation in the research (ICH E6 Guidelines for GCP). Any medical condition that is present at the time that the patient is screened will be considered as medical history and not recorded as an AE; however, if the condition worsens at any time during the study, it will be recorded and reported as an AE.

##
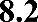
Adverse Event Reporting

All AEs that occur after any patient has been enrolled, before treatment, during treatment, or within 7 days following the cessation of treatment, whether or not they are related to the study, must be recorded on forms created for the study by the CRO.

All SAEs (related and unrelated) will be recorded from the signing of consent form or first administration of study drug until end of follow up periods. Any SAEs considered possibly or probably related to the investigational product and discovered by an investigator at any time after the study should be reported. All SAEs must be reported to the Medical Monitor within one business day of the first awareness of the event. The investigators must complete, sign and date the SAE pages, verify the accuracy of the information recorded on the SAE pages with the corresponding source documents, and send a copy by fax or email to the Medical Monitor.

Additional follow-up information, if required or available, should all be emailed or faxed to the Medical Monitor within one business day of receipt, and this should be completed on a follow- up SAE form and placed with the original SAE information and kept with the appropriate section of the CRF and/or study file.

The investigators are also responsible for reporting all SAEs to the appropriate IRB in accordance with local laws and regulations. The investigators are responsible for maintaining documentation in the study file that indicates the IRB has been properly notified. Under 21 CFR 312.32(c), the sponsor is required to notify FDA and all participating investigators in an IND safety report (i.e., 7- or 15-day expedited report) of potentially serious risks from clinical trials or any other source as soon as possible, but no later than 15 calendar days after the sponsor receives the safety information and determines that the information qualifies for reporting. Participating investigators include all investigators to whom the sponsor is providing drug under any of its INDs or under any investigator’s IND (21 CFR 312.32(c)(1)).

All AEs occurring during this clinical study will be recorded by the investigators on the appropriate eCRF in precise medical terms, along with the date and time of onset and the date and time of resolution. To avoid vague, ambiguous, or colloquial expressions, the AE should be recorded in standard medical terminology. Whenever possible, the investigators should

combine signs and symptoms into a single term that constitutes a single diagnosis. Each AE is to be evaluated for duration, severity, seriousness, and relatedness to VBI-S. The severity of the AE and its relationship to the VBI-S will be assessed by the investigators.

The investigators will treat participants experiencing AEs appropriately and observe them at suitable intervals until their symptoms resolve or their status stabilizes. If any medication is administered in response to the AE, this medication should be noted on the concomitant medication eCRF as a concomitant medication administered. The action taken and the outcome must also be recorded. The investigators will follow a non-serious AE until resolution, stabilization of the Follow-up Visit. The investigators will follow an SAE (regardless of relationship to VBI-S until the event resolves, stabilizes, or becomes non-serious. The terms of AE resolution (i.e., recovered/resolved, not recovered/not resolved, recovered/resolved with sequelae, recovering/resolving, fatal, unknown) should also be recorded.

##
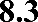
Serious Adverse Event (SAE)

An AE is considered “serious” if, in the view of either an investigator or the Sponsor, it results in any of the following outcomes (21 CFR 312.32(a)):

- Death: A death that occurs during the study or that comes to the attention of the investigator during the protocol-defined follow-up period must be reported to the Sponsor whether it is considered treatment related or not.
- A life-threatening event: An AE or suspected adverse reaction (SAR) is considered “life- threatening” if, in the view of either the investigator or the Sponsor, its occurrence places the participant at immediate risk of death. It does not include an AE or SAR that, had it occurred in a more severe form, might have caused death.
- Inpatient hospitalization or prolongation of existing hospitalization.
- Persistent or significant incapacity or substantial disruption of the ability to conduct normal life functions.
- An important medical event that may not result in death, be life threatening, or require hospitalization may be considered an SAE when, based on appropriate medical judgment, it may jeopardize the participant and may require medical or surgical intervention to prevent one of the outcomes listed above. Examples of such medical events include allergic bronchospasm requiring intensive treatment in an emergency room or at home, blood dyscrasias or convulsions that do not result in inpatient hospitalization, or the development of investigational product dependency or abuse.
- Congenital anomaly or birth defect.

If an event meets any of the above definitions, regardless of the severity or relationship of the event to the study product, the event must be reported to the Sponsor as described in Section 12.2.

Adverse events reported from clinical studies associated with hospitalization or prolongation of hospitalization are considered serious. Any hospitalization except observational admissions of less than 24 hours meets these criteria. This category also includes transfer within the hospital to an acute/intensive care unit (e.g., from a standard of care unit to an acute/intensive care unit).

Hospitalization does not include the following:

- Rehabilitation facilities, hospice facilities or respite care (e.g. caregiver relief)
- Nursing homes or skilled nursing facilities
- Emergency room visits
- Same day surgeries (as outpatient/same day/ambulatory procedures)
- <24 hour admissions for observation or evaluation

Hospitalization or prolongation of hospitalization in the absence of a precipitating, clinical AE is not in itself an SAE. Examples include:

- Admission for treatment of a preexisting condition that did not worsen
- Protocol-specified admission (e.g. for a procedure required by the study protocol)
- Hospitalizations for cosmetic elective surgery, social, and/or convenience admissions
- Pre-planned treatments or surgical procedures should be noted in the baseline documentation for the individual patient.
- Diagnostic and therapeutic procedures, such as surgery, should not be reported as AEs; however, the medical condition for which the procedure was performed should be reported if it occurs during the reporting period and meets the definition of an AE. For example, an acute appendicitis that begins during the AE reporting period should be reported as an AE, and the resulting appendectomy should be recorded as treatment of the AE.

All SAEs that occur after any patient has been enrolled, before treatment, during treatment, or within 30 days following the cessation of treatment, whether or not they are related to the study, must be recorded on forms provided by the CRO.

##
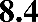
Adverse Drug Reaction (ADR) and Suspected Adverse Reaction (SAR)

An adverse drug reaction (ADR) means any AE caused by VBI-S.

Suspected adverse reaction (SAR) means any AE for which there is a reasonable possibility that the VBI-S caused the AE. For the purposes of safety reporting, “reasonable possibility” means there is evidence to suggest a causal relationship between the VBI-S and the AE. An SAR implies a lesser degree of certainty about causality than an ADR (21 CFR 312.32(a)).

## Unexpected Adverse Reaction (UAR)

The Sponsor is responsible for assessing AEs for expectedness. With regards to reporting to the Health Authority, an AE is considered “unexpected” when its nature (specificity), severity, or rate of occurrence is not consistent with applicable product information as described in the safety information provided in the protocol/package insert/investigator’s brochure/prescribing information for VBI-S. "Unexpected," as used in this definition, also refers to AEs or SARs that are mentioned in the investigator’s brochure as occurring with a class of VBI-S or as anticipated from the pharmacological properties of the VBI-S but are not specifically mentioned as occurring with VBI-S under investigation (21 CFR 312.32(a))

## Expected/ Anticipated Events

The expected/ anticipated adverse reactions observed can be separated into two classes:

1. Those more frequently encountered are due: either to contamination of the intravenous catheter and result in sepsis, or to vein irritation by concurrently infused hypertonic solutions and may result in thrombophlebitis. These adverse reactions are inseparable from the hyperalimentation procedure with or without lipid emulsion.
2. Less frequent reactions more directly related to lipid emulsions are:
   1. immediate or early adverse reactions, each of which has been reported to occur in clinical trials, in an incidence of less than 1%; dyspnea, cyanosis, allergic reactions, hyperlipemia, hypercoagulability, nausea, vomiting, headache, flushing, increase in temperature, sweating, sleepiness, pain in the chest and back, slight pressure over the eyes, dizziness, and irritation at the site of infusion, and, rarely, thrombocytopenia in neonates;
   2. delayed adverse reactions such as hepatomegaly, jaundice due to central lobular cholestasis, splenomegaly, thrombocytopenia, leukopenia, transient increases in liver function tests, and overloading syndrome (focal seizures, fever, leukocytosis, hepatomegaly. Splenomegaly and shock). The deposition of a brown pigmentation in the reticuloendothelial system, the so called “intravenous fat pigment,” has been reported in patients infused with intralipid^®^ 20%. The causes and significance of this phenomenon are unknown ([Kabi F, 2015](#_bookmark138)).

# RELATIONSHIP TO STUDY DRUG

AEs will be assigned a relationship (causality) to the study treatment (VBI-S). The investigators will be responsible for determining the relationship between an AE and the study treatment. The type of event, organ system affected, and timing of onset of the event will be factors in assessing the likelihood that an AE is related to the study treatment. Relationship of AEs to study treatment will be classified as follows:

**Definitely related:** This category applies to those AEs that the investigators believes are incontrovertibly related to the study treatment. An AE may be assigned an attribution of definitely related if or when it meets all of the following criteria: (1) it follows a reasonable temporal sequence from administration of the study treatment; (2) it could not be reasonably explained by the known characteristics of the patient’s clinical state, environmental or toxic factors, or other modes of therapy administered to the patient; (3) it follows a known response pattern to treatment with the study treatment.

**Probably related:** This category applies to those AEs which, after careful medical consideration at the time they are evaluated, are felt with a high degree of certainty to be related to the study treatment. An AE may be considered probable if or when (must have three): (1) it follows a reasonable temporal sequence from administration of the study treatment. (2) It could not readily have been produced by patient’s clinical state, environmental or toxic factors, or other therapies administered to the patient. (3) Disappears or is decreased upon discontinuation of the study treatment. (4) It follows a known response pattern to treatment with the study treatment.

**Possibly related:** This category applies to those AEs which, after careful medical consideration at the time they are evaluated, are judged unlikely but cannot be ruled out with certainty to the study treatment. An AE may be considered possible if or when (must have two): (1) it follows a reasonable temporal sequence from administration of the study treatment. (2) It could not readily have been produced by patient’s clinical state, environmental or toxic factors, or other therapies administered to the patient. (3) Disappears or is decreased upon discontinuation of the study treatment. (4) It follows a known response pattern to treatment with the study treatment.

**Unlikely related:** In general, this category can be considered applicable to those AEs which, after careful medical consideration at the time they are evaluated, are judged likely to be unrelated to the study treatment. An AE may be considered unlikely if or when (must have two): (1) it does not follow a reasonable temporal sequence from administration of the study treatment. (2) It could not readily have been produced by patient’s clinical state, environmental or toxic factors, or other therapies administered to the patient. (3) Disappears or is decreased upon discontinuation of the study treatment. (4) It does not follow a known response pattern to treatment with the study treatment.

**Unrelated:** This category applies to those AEs which, after careful consideration at the time they are evaluated, are clearly and incontrovertibly due to extraneous causes (disease, environment, etc.) and determined with certainty to have no relationship to the study treatment.

## Recording Adverse Events

Adverse events revealed by observation will be recorded during the study at the investigational site. Clinically significant changes in laboratory values, blood pressure, and pulse need not be reported as AEs. However, abnormal values that constitute an SAE or lead to discontinuation of administration of study drug must be reported and recorded as an AE. Information about AEs will be collected from the signing of consent form or first administration of VBI-S until the end of the study. Serious Adverse Event information will be collected from signing of the informed consent form until the last study visit. The AE term should be reported in standard medical terminology when possible. For each AE, the reporting investigator will evaluate and report the onset (date and time), resolution (date and time), severity, action taken, seriousness and outcome (if applicable), and whether or not it caused the patient to discontinue the study.

The guidelines outlined in National Cancer Institute’s *Common Terminology Criteria for Adverse Events Version 5.0*. (NCI-CTCAE) v5.0) will be used for assessing the intensity of the event. The general guidelines for assessing the AE grade appear below. Full guidelines obtained at https://ctep.cancer.gov/protocolDevelopment/electronic_applications/ctc.htm#ctc_50.

Please refer to the following standards in the NCI-CTCAE manual:

- Grade 1 = mild; asymptomatic or mild symptoms; clinical or diagnostic observations only; intervention not indicated.
- Grade 2 = moderate minimal, local, or noninvasive intervention indicated, limiting age- appropriate instrumental activities of daily living (ADL) e.g., preparing meals, shopping for groceries or clothes, using the telephone, managing money, etc.
- Grade 3 = severe or medically significant but not immediately life-threatening; hospitalization or prolongation of hospitalization indicated; disabling; limiting self-care ADL (e.g., bathing, dressing, and undressing, feeding self, using the toilet, taking medications, and not bedridden).
- Grade 4 = life-threatening consequences; urgent intervention indicated.
- Grade 5 = death related to AE.

## Treatment Given as a Result of the Event

The event impact in terms of treatment provided will be as either: none, medication administered, non-drug therapy administered, surgery performed, hospitalization, or other (with a specification).

## Outcome Assessment

The outcome of the event will be assessed as either: resolved, resolved with sequelae, ongoing, or death. Only one AE per patient is allowed to have an outcome assessment as “death.” If there are multiple causes of death for a given patient, only the primary cause of death will have an outcome of death.

## SAE Follow-up

All patients experiencing an SAE, including the discontinued patients, must be closely followed until sufficient information is obtained to indicate a return to normal status or until the event stabilizes at a level acceptable to the investigator (i.e., recovery, return to baseline status, no further improvement expected, or death). For each SAE indicated as an unresolved event on the initial report, regardless of whether the patient completed the study or withdrew, the site should submit a follow-up report with updated information.

# STATISTICAL ANAYLSIS

This section provides the basis for the statistical analysis plan (SAP) for the study. The SAP may be revised during the study to accommodate amendments to the clinical study protocol and to make changes to adapt to unexpected issues in study execution and data that may affect the planned analyses. The final SAP will be issued before the database is locked. Additional analysis may be performed if deemed necessary. Any deviations from the planned analysis will be described and justified in a separate document and in the clinical study report.

## Treatment Groups

This is a single arm open-label study and only one (1) treatment group with VBI-S will be assessed for efficacy and safety of VBI-S in this study.

## Sample Size Consideration

The sample size is based on a hypothesized rate of achievement of the primary and secondary endpoint efficacy end point of 80%.

## General Statistical Considerations

The statistical analysis will be performed using SAS^®^ version 9.4 or higher. Descriptive statistics with 95% confidence intervals will be used to summarize data according to treatment group. Change in blood pressure to a hypothetical null result using an unpaired two tailed Student’s t test. P< 0.05 will be considered significant. All secondary and exploratory endpoints will be analyzed if the primary endpoint is met. The Kaplan – Meier Estimator will be used to describe patient survival.

## Analysis Population

The following populations will be used for analysis:

- - - - Safety Analysis Set: all patients who have received any amount of VBI-S.
      - Intent to treat (ITT) population: all patients who have received any amount of VBI-S.

## Missing Data

For the primary/secondary outcome evaluation appropriate methods will be used to handle any missing data. The details of techniques for handling of missing data will be included in the SAP for the study which will be finalized prior to database lock.

## Analysis Methods

A formal detailed statistical analysis plan (SAP) will be created prior to the review of any data

## Interim Analysis

Once the first 10 patients complete the day 28 assessment, an interim analysis of the data will

be performed and presented to the Data Safety Monitoring Board (DSMB). Based on their assessment the DSMB will recommend whether to continue to trial as planned, modified or discontinued

## Safety Evaluation

Safety will be monitored over the course of the study by the DSMB.

Safety evaluations will be conducted at specified time points throughout the study, in patients who continue treatment, every 7 days or if clinically indicated thereafter. These evaluations will include a targeted physical/neurological examination, electrocardiography in patients where clinically indicated and clinical laboratory studies, including hematology, blood chemistries and urinalysis.

Treatment-emergent adverse events (TEAEs) will be documented if 1) an adverse event which starts after the first dose of VBI-S, or 2) an adverse event documented during the pre-infusion period which increases in severity after the first dose of VBI-S.

The frequency and incidence of TEAEs will be summarized by system organ class (SOC) and preferred term (PT).

Following summaries will be presented:

Summary of TEAEs by SOC and PT

Summary of TEAEs by SOC, PT, number and severity of event Summary of TEAEs by SOC, PT, maximum study drug relationship

Summary of TEAEs leading to discontinuation of study drug by SOC and PT Summary of TEAEs leading to death by SOC and PT

Summary of TEAEs related to study drug by SOC and PT Summary of TEAEs related to study drug by SOC, PT, and severity; Summary of serious TEAEs by SOC and PT;

Summary of serious TEAEs related to study drug by SOC and PT.

Data collected from other safety evaluations will be summarized descriptively and/or listed

# DIRECT ACCESS TO SOURCE DATA/ DOCUMENTS

## Study Monitoring

Before an investigational site can enter a patient into the study, a representative of CRO will visit the investigational study site to:

- Determine the adequacy of the facilities
- Discuss with the investigators and other personnel their responsibilities with regards to protocol adherence, and the responsibilities of CRO. This will be documented in a Trial Master File (TMF).

During the study, a monitor from the CRO will have regular contacts with the investigational site, for the following:

- Provide information and support to the investigators
- Confirm that facilities remain acceptable
- Confirm that the investigational team is adhering to the protocol, that data are being accurately recorded in the case report forms, and that investigational product accountability checks are being performed
- Perform source data verification. This includes a comparison of the data in the case report forms with the patient’s medical records at the hospital or practice, and other records relevant to the study. This will require direct access to all original records for each patient (e.g. clinic charts).
- Record and report any protocol deviations not previously sent to the CRO.
- Confirm AEs and SAEs have been properly documented on CRFs and confirm any SAEs have been forwarded to the CRO and those SAEs that met criteria for reporting have been forwarded to the IRB.

The monitor will be available between visits if the investigators or other staff needs information or advice.

## Audits and Inspections

Authorized representatives of VivacelleBio, a regulatory authority, an Independent Ethics Committee or an Institutional Review Board may visit the site to perform audits or inspections, including source data verification. The purpose of a VivacelleBio audit or inspection is to systematically and independently examine all study-related activities and documents to determine whether these activities were conducted, and data were recorded, analyzed, and accurately reported according to the protocol, Good Clinical Practice guidelines of the International Conference on Harmonization, and any applicable regulatory requirements. The investigators should contact VivacelleBio immediately if contacted by a regulatory agency

about an inspection.

# INSTITUTIONAL REVIEW BOARD

The investigators at a site will provide the Institutional Review Board/Independent Ethics Committee (IRB/IEC) with all appropriate documents and updates as required by their IRB/IEC and in agreement with policy established by the Sponsor, including but not limited to the clinical study protocol, informed consent form, and any advertising materials. The study will not be initiated until the IRB/IEC provides written approval of the aforementioned documents and until approval documents have been obtained. . The investigators are required to maintain an accurate and complete record of all written correspondence to and received from the IRB/IEC and must agree to share all such documents and reports with the Sponsor.

No changes from the final approved protocol will be initiated without the IRB/IEC’s prior written approval or favorable opinion of a written amendment, except when necessary to eliminate immediate hazards to the patients or when the modification does not involve the patient’s participation in the trial.

## Investigator’s Responsibilities

The investigators are responsible for performing the study in full accordance with the protocol and the current revision of the Declaration of Helsinki, the Good Clinical Practice: Consolidated.

## Patient Informed Consent Requirements

Written and oral information about the study in a language understandable by the patient or LAR will be given to all patient’s or LARs by an Investigator and/or designee. Written informed consent will be obtained before any procedures or assessments that would not otherwise be required for the care of the patient are done and after the aims, methods, anticipated benefits, potential hazards, and insurance arrangements in force are explained and the patient has been given sufficient time to ask questions and consider participation in the study. It will also be explained that the patient is free to refuse entry into the study and free to withdraw the represented from the study at any time without prejudice to future treatment. It is permissible for a https://clincalc.com/IcuMortality/SOFA.aspx third person (e.g., a family member) to be present during the explanation of the study.

The written Informed Consent Form ICF is to be in compliance with CFR 21 Part 50.27 and GCP guidelines. The Sponsor and/or designated CRO will approve the ICF and all amendments to the ICF prior to submission to the IRB/IEC. A copy of the ICF to be used will be submitted by the investigators to their respective IRB/IEC for review and approval prior to the start of the study. Each study site must provide the Sponsor with an unsigned copy of IRB/IEC-approved ICF along with applicable documentation to support this approval. The original signed ICF is retained in the patient's study records, and a copy is provided to the patient. A second copy may be filed in the patient’s medical record, if allowed by institutional policy.

# QUALITY CONTROL AND QUALITY ASSURANCE

To ensure compliance with Good Clinical Practices and all applicable regulatory requirements, Vivacelle may conduct a quality assurance audit. Please see [Section 10.2](#_bookmark110) for more details regarding the audit process.

## Monitoring requirements

To fulfill the obligations outlined in 21 Code of Federal Regulations (CFR) Part 312 and ICH guidelines, which requires the Sponsor to maintain current personal knowledge of the progress of a study, the Sponsor's designated monitor will visit the center(s) during the study as well as maintain frequent telephone and written communication. The investigators will permit the Sponsor to monitor the study as frequently as is deemed necessary and provide access to medical records to ensure that data are being recorded adequately, that data are verifiable and that protocol adherence is satisfactory. The investigators will permit representatives of the Sponsor and/or designated CRO to inspect all eCRFs and corresponding study patient original medical records (source documents) at regular intervals throughout the study. Patient original medical records and other relevant data must be available to support all data recorded in the eCRF. In addition to the original medical records, these data may include but are not limited to, study, laboratory and diagnostic reports, ulcer images, quality of life questionnaire, etc. Site inspections serve to verify strict adherence to the protocol and the accuracy of the data being entered on the case report forms, in accordance with federal regulations. A Monitoring Log will be maintained at each study site which the monitor will sign, date and state the type of visit. The investigators should be aware that the study site and patient records may be inspected by the Sponsor and or representatives of the designated CRO, FDA or other regional regulatory authority.

## Acceptability of electronic Case Report Forms (eCRFs)

Electronic CRFs must be completed for each patient who has signed an informed consent form. For patients who are screen failures, this would be limited to the screen failure eCRF page. All source documents and eCRFs will be completed as soon as possible after the patient's visit. Corrections to data on the eCRFs will be documented. The investigators will review eCRFs to indicate that, to his/her knowledge, they are complete and accurate. Electronic CRFs will be reviewed by the Sponsor’s or designated CRO’s monitor, who will make a decision as to their acceptability.

## Modification of Protocol

The investigators will not modify or alter this protocol without first obtaining the concurrence of the Sponsor. Approval by the Investigator’s IRB must also be obtained prior to implementation of the change, with two exceptions:

- When necessary to eliminate apparent immediate hazard to the patient; OR
- When the modification does not involve the patient’s participation in the trial.

An amendment may also require modification of the informed consent form. The investigators will provide an approval letter for the amendment and revised informed consent form, if applicable, to the Sponsor. An amendment must be provided in writing, and it must be dated by

both the Sponsor and the Investigator. If necessary, the Sponsor will submit protocol amendments to FDA and other appropriate regulatory authorities and notify other Investigators using this protocol.

## Reporting Protocol Deviations

The investigators are obligated to follow the protocol without departure from the requirements written in the protocol. If the investigators deviates from the protocol requirements, the Sponsor will make the determination as to whether the patient will continue in the study. The Sponsor also has the right to discontinue the patient for protocol violations. The IRB may also have to be contacted if safety to the patient or if the scientific soundness of the study is involved. All protocol deviations must be documented in the eCRFs.

## Major Protocol Deviation and Violation

A major protocol deviation or violation is a deviation from the IRB approved protocol that may affect the patient's rights, safety, or well-being and/or the completeness, accuracy and reliability of the study data. Examples of this include:

- Failure to obtain informed consent prior to initiation of study-related procedures
- A research patient does not meet the protocol's eligibility criteria but was enrolled without prior approval from the sponsor.
- A research patient received the wrong treatment or incorrect dose.
- A research patient met withdrawal criteria during the study but was not withdrawn.
- A research patient received a prohibited concomitant medication.
- Failure to treat research patients per protocol procedures that specifically relate to primary efficacy outcomes.
- Changing the protocol without prior sponsor and IRB approval.
- Multiple minor violations of the same nature after multiple warnings.

## Minor Protocol Deviation and Violation

A minor protocol deviation is any change, divergence, or departure from the study design or procedures of a research protocol that has not been approved by the IRB and which DOES NOT have a major impact on the patient's rights, safety or well-being, or the completeness, accuracy and reliability of the study data.

Examples of this include:

- Study visits occurred outside the protocol required time frame because of the participant’s schedule.
- Blood samples obtained at times close to but not precisely at the time points specified in the protocol.

# ETHICS

## Ethics Review

The final study protocol, including the final version of the Informed Consent Form, must be approved or given a favorable opinion in writing by an IRB or IEC as appropriate.

The Principal Investigator is responsible for informing the IRB or IEC of any amendment to the protocol in accordance with local requirements. In addition, the IRB or IEC must approve all advertising used to recruit patients for the study. The protocol must be re-approved by the IRB or IEC upon receipt of amendments and annually, as local regulations require.

The Principal Investigator is also responsible for providing the IRB with reports of any reportable serious adverse drug reactions. Progress reports and notifications of serious adverse drug reactions will be provided to the IRB or IEC according to local regulations and guidelines.

## Ethical Conduct of the Study

The study will be performed in accordance with ICH/Good Clinical Practice, and applicable regulatory requirements.

## Written Informed Consent

The investigators at each center will ensure that the patient or their LAR is given full and adequate oral and written information about the nature, purpose, possible risk and benefit of the study. A patient or their LAR must also be notified that they are free to discontinue the treated patient from the study at any time. The patient or their LAR should be given the opportunity to ask questions and allowed time to consider the information provided.

The patient or their LAR’s signed and dated informed consent must be obtained before conducting any study procedures.

The investigators must maintain the original, signed Informed Consent Form. A copy of the signed Informed Consent Form must be given to the LAR or patient.

# DATA HANDLING AND RECORDKEEPING

## Recording and Collection of Data

The primary source document for this study will be the patient's medical record. If separate research records are maintained by the investigators, the medical record and the research records will be considered the source documents for the purposes of auditing the study.

Applicable source data will be manually transcribed to approved case report forms (CRF). The investigators are ultimately responsible for the accuracy of the data transcribed on the forms. All source documents and CRFs will be completed as soon as possible after the patient's visit.

The investigators will review CRFs to indicate that, to his/her knowledge, they are complete and accurate. Designated source documents will be signed and dated by the appropriate study personnel. The investigators must agree to complete and maintain source documents and CRFs for each patient participating in the study.

All research data will be entered, either electronically or manually, into a computerized database. The clinical database will be designed by the clinical data manager in accordance with 21 CFR Part 11 and based on protocol requirements defined by the Sponsor in association with the Principal Investigator.

The investigators will maintain a confidential list of study patients that will include each patient’s study number, name, date of birth, and unique hospital identification number if applicable. This list will be kept by the investigators and will not be collected by the Sponsor. A notation will be made in the patient’s case history/medical chart that he/she is participating in a clinical study and has provided a signed and dated ICF as well as a release for protected health information as required by local policies. The investigators must also maintain a separate screening log of all the patients screened for participation in the study; it should include gender, age, eligibility status, reason for ineligibility, if applicable; and study allocated patient number, if applicable.

## Clinical Data Management

The Sponsor and/or designated CRO will be responsible for the processing and quality control of the data. Data management will be carried out as described in the Sponsor’s or CRO’s standard operating procedures (SOPs) for clinical studies. The handling of data, including data quality control, will comply with regulatory guidelines (e.g., ICH E6 GCP, and local regulations where applicable) and the Sponsor’s or the CRO’s SOPs as well as provisions of the study- specific Data Management Plan.

## Inspection of Records

Vivacelle Bio will be allowed to conduct site visits to the investigation facilities for the purpose of monitoring any aspect of the study. The investigators agree to allow the monitor to inspect the drug storage area, study drug stocks, drug accountability records, patient charts and study source documents, and other records relative to study conduct.

## Retention of Records

All study documentation at the Investigator’s site and Sponsor site will be archived in accordance with ICH GCP E6 and the Sponsor’s quality standards and SOPs.

The investigators will maintain all research records, reports, and case history reports for a period of two (2) years after regulatory approval of the investigational product. If no application is filed or if the application is not approved, records must be maintained for two (2) years after all investigations have been completed, terminated, or discontinued and the FDA has been notified.

These documents should be retained for a longer period however, if required by the applicable regulatory requirements or if needed by Sponsor or its authorized representative (as per GCP 5.5.11). At the completion of the study, details of the archival process must be provided to the Sponsor. Study records are patient to inspection by applicable health and regulatory agencies at any time.

Records to be retained by the investigators include, but are not restricted to:

- Source data and the primary records upon which they are based (e.g., patient’s progress notes, adverse event data, test results, and any other diagnostic procedures required to evaluate the progress of the study)
- Completed CRFs
- Signed protocols and protocol amendments
- Laboratory results, ranges, and certifications
- IP and accountability records
- Study personnel signature log
- Monitoring logs
- Correspondence to and from the Sponsor, designee and IRB
- Investigator and sub-investigator CVs
- Signed informed consent and protected health information consent forms
- Patient screening
- SAE reports
- IRB approval and re-approval letters
- Completed study assessments and questionnaires
- Other documents pertaining to the conduct of the study

These documents must be maintained and kept on file by the investigators so that the conduct of the study can be fully documented and monitored. At the completion of the study, details of the archival process must be provided to the Sponsor. Study records should not be transferred from site or destroyed without prior written agreement between the Sponsor and the study investigators. Study records are patient to inspection by applicable health and regulatory agencies at any time.

# PUBLICATION POLICY

All information supplied by VivacelleBio in connection with this study and not previously published, is considered confidential information. This information includes, but is not limited to, the Investigator’s Brochure, clinical protocol, case report forms and other scientific data. Any data collected during the study are also considered confidential. This confidential information shall not be disclosed to others without prior information to VivacelleBio and shall not be used except in the performance of this study.

It is understood by the investigators that VivacelleBio will use the information collected in this clinical trial in connection with further development of VBI-S. Therefore, this information may be disclosed as required to other Investigators or appropriate regulatory authorities. By agreeing to participate in this clinical trial, the investigators understands that he/she has an obligation to provide VivacelleBio with complete test results and all data developed during this trial.

# LIST OF REFERENCES

- 1. Agarwal, H.S., Taylor, M.B., Grzeszczak, M.J. et al. Extra corporeal membrane oxygenation and plasmapheresis for pulmonary hemorrhage in microscopic polyangiitis. *Pediatr Nephrol*. 2005; 20: 526.
  2. Armbruster M, Grimley E, Rodriguez J, et al. Soybean oil: a potentially new intravascular perfusate. *Perfusion*. 2013;28(2):160-166.
  3. Brand DA, Patrick PA, Berger JT, et al. Intensity of Vasopressor Therapy for Septic Shock and the Risk of In-Hospital Death. *J Pain Symptom Manage*. 2017;53(5):938- 943.
  4. Centers for Disease Control and Prevention. Sepsis: Data & Reports. Last updated: August 23, 2016. <https://www.cdc.gov/sepsis/datareports/index.html>. Accessed on:

April 15, 2019.

- 1. Ciechanowicz S, Patil V. Lipid emulsion for local anesthetic systemic toxicity. *Anesthesiol Res Pract*. 2011;2012:131784.
  2. Clemente TE, Cahoon EB. Soybean oil: genetic approaches for modification of functionality and total content. *Plant Physiol*. 2009;151(3):1030–1040.
  3. Girardin E, Dayer JM. Cytokines and antagonists in septic shock. Schweiz Med Wochenschr 1993; 123(11):480-491
  4. Hansen LM, Hardie BS, Hidalgo J. Fat emulsion for intravenous administration: clinical experience with intralipid 10%. *Ann Surg*. 1976;184(1):80–88.
  5. Ikeda Y, Mochizuki Y, Matsumoto H, et al. L-histidine but not D-histidine attenuates brain edema following cryogenic injury in rats. *Acta Neurochir Suppl*. 2000;76:195-7.
  6. Kabi F. Intralipid 20% A 20% I.V. Fat Emulsion Package Insert. 2015.
  7. Kabi F. Intralipid Lipid Injectable Emulsion, FK Std Soybean Oil 10%, 20%, 30% w.v. Product Monograph 2017.
  8. Mayr FB, Yende S, Angus DC. Epidemiology of severe sepsis. *Virulence*. 2013;5(1):4- 11.
  9. Méjean M, Brunelle A, Touboul D. Quantification of tocopherols and tocotrienols in soybean oil by supercritical-fluid chromatography coupled to high-resolution mass spectrometry. *Anal and Bioanal Chem*. 2015;407(17):5133-5142.
  10. Pontes-Arruda A, Liu FX, Turpin RS, et al. Bloodstream infections in patients receiving manufactured parenteral nutrition with vs without lipids: is the use of lipids really deleterious? *JPEN J Parenter Enteral Nutr*. 2012;36(4):421-30.
  11. Schumer W. Pathophysiology and treatment of septic shock. *Am J Emerg Med*. 1984;2(1):74-7
  12. National Institute of General Medical Sciences: Sepsis. Content Updated: January 2018. [https://www.nigms.nih.gov/Education/Pages/factsheet_sepsis.aspx#1.](https://www.nigms.nih.gov/Education/Pages/factsheet_sepsis.aspx#1) Accessed: March 27, 2019.
  13. Simpkins CO, Ekshyyan V, Snyder B. Histidine inhibits the degradation of cells suspended in ringer's lactate. *J Trauma*. 2007;63(3):565-572.
  14. Singh U, Devaraj S. Vitamin E: inflammation and atherosclerosis. *Vitam Horm*. 2007;76:519-49.
  15. Turpin RS, Canada T, Liu FX, Mercaldi CJ, Pontes-Arruda A, Wischmeyer P. Nutrition therapy cost analysis in the US: pre-mixed multi-chamber bag vs compounded parenteral nutrition. *Appl Health Econ Health Policy*. 2012;9(5):281–292.
  16. Weinberg GL. Lipid emulsion infusion: resuscitation for local anesthetic and other drug overdose. *Anesthesiology*.2012;117(1):180–187.

# 
